# Investigation and design of the dual specificity of the PRDM9 protein lysine methyltransferase

**DOI:** 10.1038/s42003-025-08207-4

**Published:** 2025-05-29

**Authors:** Dimitri Graf, Philipp Schnee, Jürgen Pleiss, Sara Weirich, Albert Jeltsch

**Affiliations:** Institute of Biochemistry, University of Stuttgart, Stuttgart, Germany

**Keywords:** Enzyme mechanisms, Epigenetics, Methylases, Histone post-translational modifications, Transferases

## Abstract

The PRDM9 protein lysine methyltransferase is essential in meiotic recombination where it trimethylates H3K4 and H3K36 in chromatin. However, it is not known how this enzyme can specifically methylate these two substrates despite their dissimilar amino acid sequences. Using biochemical and molecular dynamics simulation approaches, we uncover that PRDM’s unique dual substrate specificity is based on distinct interaction modes of the enzyme with both substrates. Our data show that PRDM9 interacts with the H3K4 and H3K36 peptides through a bipartite peptide binding cleft, comprising one part specific for H3K4 but tolerating H3K36, and a second part with the opposite properties. Binding of the H3K4 and H3K36 peptide substrates occurs in slightly different conformations which enables the specific recognition of both substrates. While wildtype PRDM9 showed higher activity on H3K4 peptides, site-directed mutagenesis of residues involved in PRDM9-peptide contacts allowed us to strongly modulate the K4/K36 preferences creating mutants with elevated preference for H3K4, mutants with equal methylation of both substrates and even mutants with preference for H3K36. Our data illustrate evolutionary pathways to swap the sequence specificity of PKMTs by few amino acid exchanges, a process that happened several times in the divergent evolution of PKMTs.

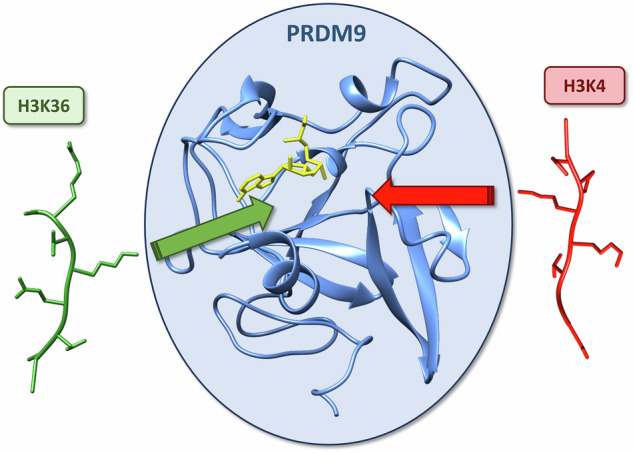

## Abstract (web summary)

Biochemical and modelling data explain how PRDM9 specifically methylates the very different H3K4 and H3K36 substrates by distinct substrate interaction modes. Our data illustrate evolutionary pathways for specificity swapping of PKMTs.

## Introduction

Histone tails are pivotal in the epigenetic regulation of chromatin states through a vast number of post-translational modifications (PTMs), including acetylation, methylation, ubiquitylation and phosphorylation^1,2^. These modifications, which occur on specific amino acids such as lysine, arginine, serine, or threonine, have a variety of critical roles in development and pathogenicity^3,4^. Among them, many lysine methylation sites with highly important roles have been identified^5^, for example H3K4^6^ and H3K36^7^. Both modifications are associated with active transcription, H3K4 trimethylation (H3K4me3) is found at active gene promoters, while H3K36me3 is enriched in the gene bodies of actively expressed genes. Downstream biological effects are mediated by specific binding proteins, so called readers, which recruit other chromatin factors to the respective genomic loci^8^. The methylation of lysine residues is catalyzed by Protein lysine methyltransferases (PKMTs), which can transfer up to 3 methyl groups from S-Adenosyl-L-methionine (AdoMet) onto the ε-amino group of lysine residues^9^. While most human PKMTs share a similar catalytic domain called SET-domain (Su(var)3–9, Enhancer-of-zeste and Trithorax)^10^, they are often specialized to methylate a specific histone target site up to a distinct level of methylation. One exception to this rule can be found in the case of PR/SET domain containing protein 9 (PRDM9)^11^. PRDM9 was first described in 2006 as a factor that is crucial for meiosis in mice and human cells^12,13^. Later, it was found to be a lysine methyltransferase which specifically up to trimethylates H3K4 and H3K36 at particular genomic sites called recombination hotspots and the activity of PRDM9 was shown to be essential for double strand break formation during meiotic recombination^14–16^.

**Fig. 1:**
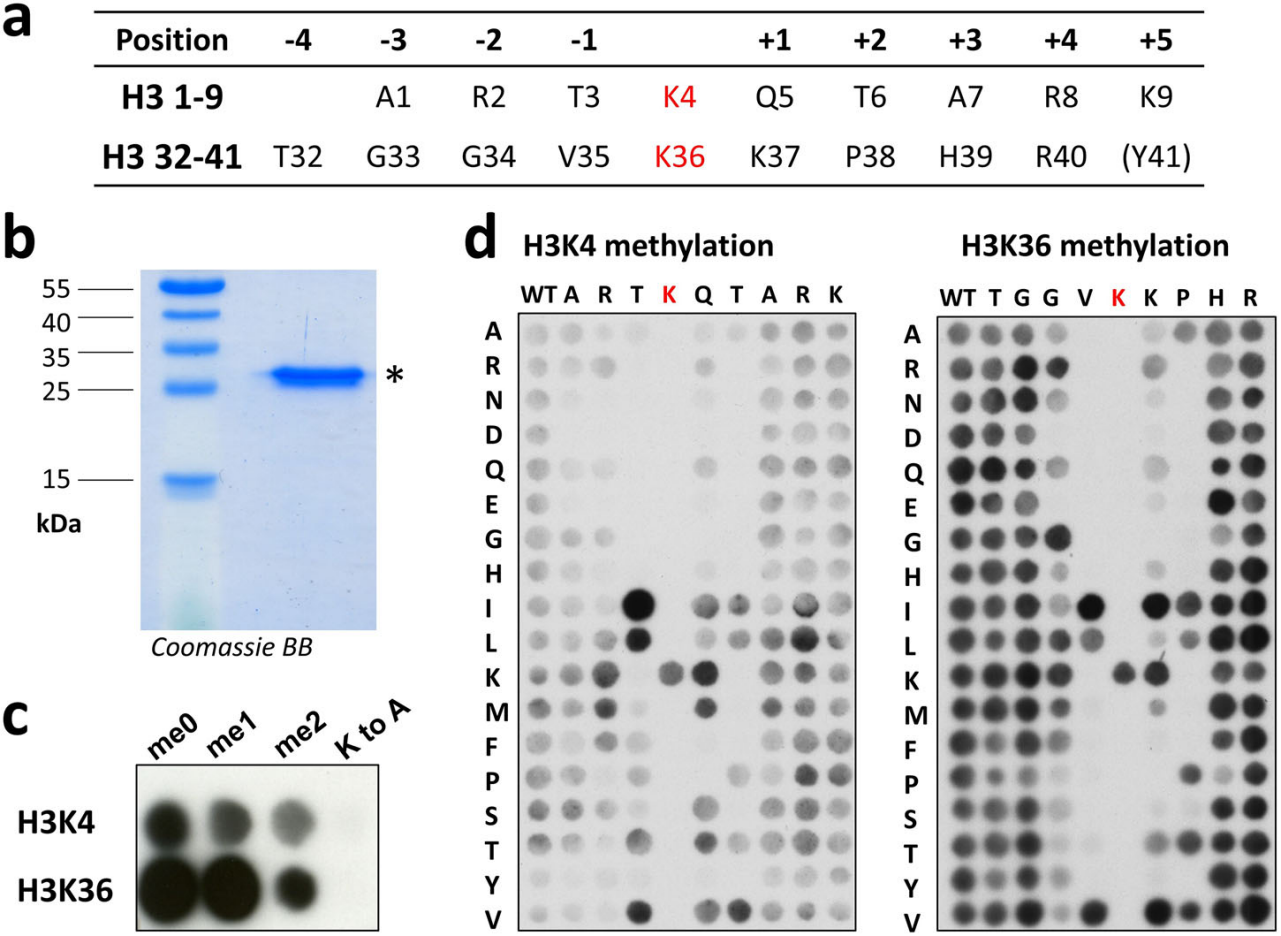
PRDM9 (195–415) purification and SPOT peptide array methylation. **a** Comparison of the amino acid sequences of positions 1–9 of H3K4 and 32–41 of H3K36. The relative position in relation to the correspondent target lysine is indicated as well. **b** SDS-gel of purified PRDM9 (195–415) stained with Coomassie Brilliant Blue. **c** Exemplary image of the autoradiography of peptide SPOT array methylation on H3 (1–15) and H3 (29–43) peptides in un-, mono- and dimethylated form by PRDM9 (195–415). Target lysine K-to-A mutant peptides were used as controls. **d** Exemplary images of the autoradiography of H3K4 and H3K36 specificity scan peptide arrays methylated by PRDM9 (195–415). Arrays of 15 aa long peptides (H3 1–15 and 29–43) were synthesized using the H3K4 and H3K36 template sequences as represented in the horizontal axis. Residue surrounding the target lysine residues were systematically exchanged against other amino acid residues as shown in the vertical axis. See also Supplementary Fig. 2a.

PRDM9 consists of an N-terminal KRAB domain which is important for protein-protein interaction, a nuclear localization signal, and the catalytic PR/SET domain, which is distantly related to the catalytic SET domain found in many other human PKMTs^17^. The C-terminal part of PRDM9 contains a highly evolvable array of zinc fingers which mediate DNA sequence-specific targeting to the recombination hotspots^18,19^. According to the current model^11^, PRDM9 binds to DNA in the meiotic prophase and deposits H3K4me3 and H3K36me3. The methylation of H3K4 is potentially recognized by the CXXC1 protein, which promotes further PRDM9 recruitment. The H3K36 trimethylation was suggested to aid in the later process of DSB repair. The dual specificity of PRDM9 for H3K4 and H3K36 is not only fascinating from its regulatory perspective, but also in molecular terms, because both target sequences do not share amino acid residues (Fig. 1a), but still both are specifically recognized by PRDM9. A crystal structure of PRDM9 with bound H3K4 peptide was solved^20^ showing that the target lysine is located in a channel formed by Trp293, Tyr357, and Tyr361 while the ε-amino group is surrounded by three tyrosine residues (Tyr357, Tyr276, and Tyr341). Moreover, it was observed that the H3K4 peptide specifically interacts with the catalytic site through hydrogen bonding between the H3Q5 and E360 of PRDM9 as well as an interaction H3T3 and A287, but also through stacking interactions between H3R2 and Y361^20^. Peptide docking experiments with H3K36 and PRDM9^14^ suggested, that the peptide positions −1 and +2 relative to the target lysine in this substrate occupy hydrophobic cavities of PRDM9. Furthermore, it was proposed that the interactions formed with the aliphatic sidechain of H3R2 are lost in the H3K36 context, and an additional electrostatic interaction between H3K36-K37 and the E360 is formed^14^. Abolishing the latter contact by mutating E360 to lysine led to a strong reduction of H3K36 methylation without affecting the H3K4 methylation in experiments performed with the PR/SET domain^21^. Mutating the glutamic acid to proline on the other hand reduced the H3K4 methylation activity and abolished H3K36 methylation^21^. However, there is still a lack of understanding of how PRDM9 manages to productively interact with both of its substrates H3K4 and H3K36, in particular as they have very different amino acid sequences (Fig. 1a).

It was the aim of this study to gain a deeper understanding of characteristic molecular interactions formed between the two substrate peptides and the catalytic site of PRDM9, which ultimately establish a dual specificity peptide recognition that is unique among PKMTs. Interestingly, PRDM9 is not closely related to other PKMTs methylating either H3K4 or H3K36. This is illustrated by the position of the respective enzymes in the phylogenetic tree of SET domain-containing PKMTs (taken from^22^) (Supplementary Fig. 1a) and the lack of conservation of peptide contacting amino acids residues of PRMD9 (Supplementary Fig. 1b, c). Hence amino acids sequence comparisons of PKMTs cannot provide clues about PRMD9’s substrate recognition. We, therefore, tackled this challenging scientific question by complementary experimental approaches to investigate PKMTs^22^ including biochemical specificity analyses of PRDM9 in the context of both substrate peptides and molecular dynamics (MD) simulations of PRDM9 with bound H3K4 and H3K36 peptides. The models and hypothesis derived from these studies were systematically challenged by the generation and investigation of 14 PRDM9 mutants. Our data show that the peptide interface of PRDM9 is not perfectly optimized for any of the two substrates. The reason for this is that PRDM9 specifically interacts with the H3K4 and H3K36 peptides through a bipartite peptide recognition cleft, comprising one part specific for the H3K4 peptide but tolerating H3K36, and a second part with preference for H3K36 but still tolerating H3K4 residues. One striking conformational distinction between the H3K4 and H3K36 peptides is observed in their N-terminal end: In the case of the H3K4 substrate, the side chain of R2 is tightly bound leading to the placement of the N-terminal end in a narrow pocket not allowing further extension of the peptide. In the case of the H3K36 peptide, G33 and G34 occupy the place of the H3K4-R2 side chain, thereby threading the end of the peptide to the surface of PRDM9 allowing continuation of the peptide chain. Site-directed mutagenesis of several residues involved in PRDM9-peptide contacts allowed us to modulate the K4/K36 preferences strongly revealing mutants with elevated preference for H3K4, mutants with equal methylation of H3K4 and H3K36 and even mutants with a preference for H3K36. These data illustrate potential pathways of molecular evolution to modulate PKMT specificity by few amino acid exchanges.

## Results

### PRDM9 expression and purification

His-tagged PRDM9 was overexpressed and purified using Ni-NTA affinity chromatography (Fig. 1b). To test the activity of the purified enzyme, SPOT peptide array methylation experiments were performed using H3K4 and H3K36 peptides in unmethylated, mono- or dimethylated form as substrates. Radioactively labeled AdoMet was employed as cofactor and the transfer of radioactivity monitored by autoradiography. As control, peptides were included in these experiments which had the target lysine mutated to an alanine (K-to-A mutants) (Fig. 1c). In agreement with literature data^14,20,23^, methylation signals were observed for H3K4 and H3K36 peptides in the unmethylated form, as well as for peptides that were already mono- or dimethylated (with declining intensity) indicating that PRDM9 can introduce up to trimethylation in both substrates. The absence of methylation of the K-to-A mutant substrate peptides demonstrates that K4 and K36 are the only target sites for PRDM9 methylation in the corresponding peptides confirming the ability of PRDM9 to modify H3K4 and H3K36 targets with sequence specificity. The molecular mechanism of the specific recognition of these two distinct sequences by one enzyme is unknown and was investigated in the next part of our study.

### PRDM9 specificity array methylation

**Fig. 2:**
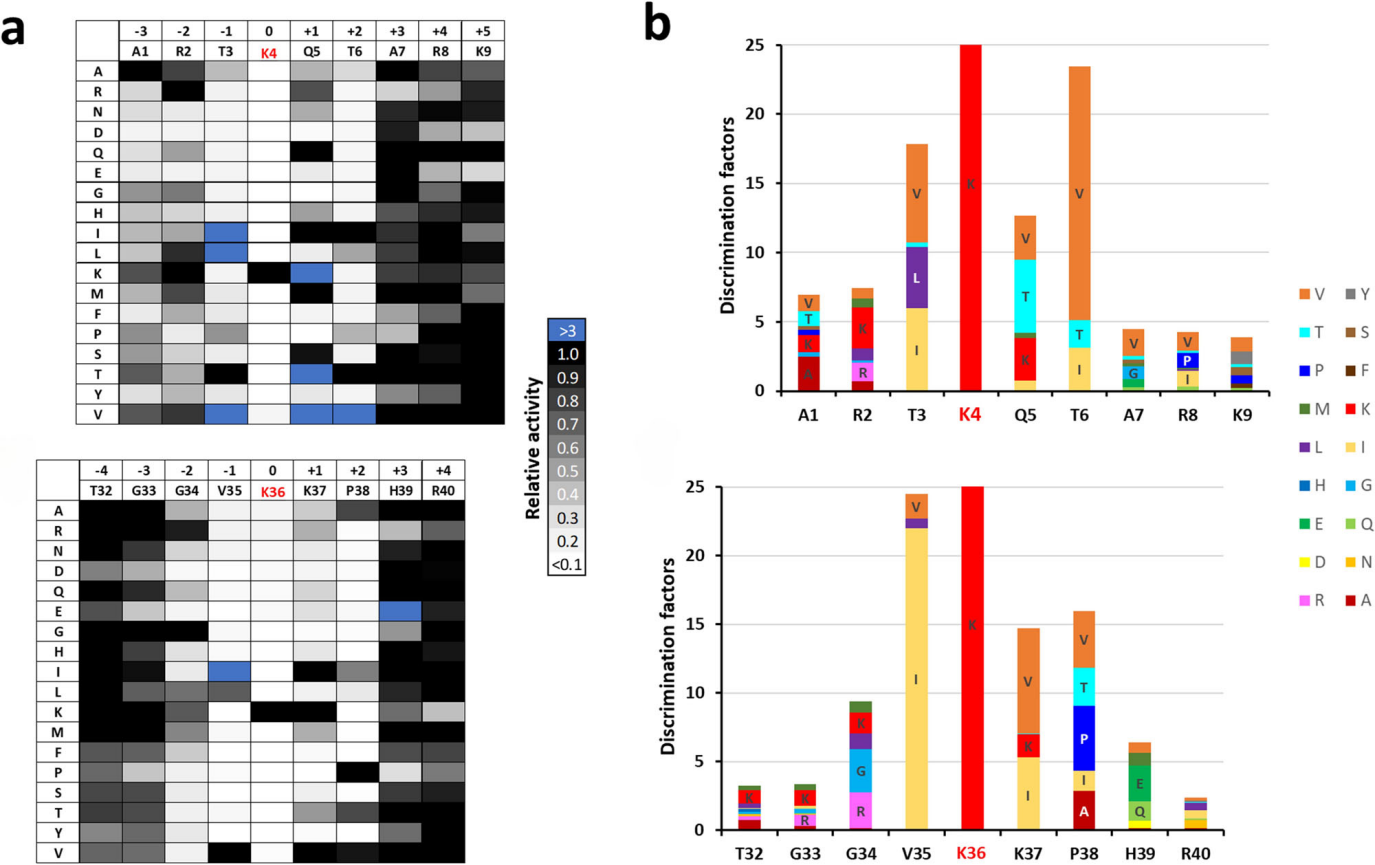
Specificities analysis of PRDM9 (195–415) in the context of the H3K4 and H3K36 peptides. **a** Averaged signal intensity profiles based on two replicates of H3K4 (upper panel) and H3K36 specificity scan peptide arrays (lower panel) after methylation by PRDM9 as shown in Fig. 1d. See also Supplementary Fig. 2b. **b** Bar chart representing the discrimination factors of any amino acid relative to all others at all positions in the H3K4 (upper panel) and H3K36 peptides (lower panel). The panels were aligned according to the target lysine residues.

To investigate the position-specific amino acid preferences of PRDM9 for methylation of the H3K4 and H3K36 substrates, we conducted peptide SPOT array methylation experiments with specificity scan peptide arrays^24,25^. To this end, peptide SPOT arrays of H3 1–15 (containing K4) and H3 29–43 (containing K36) were synthesized. In these arrays, the individual amino acid positions 1–9 in the H3K4 context (corresponding to the positions −3 to +5 relative to the target lysine) and position 32–40 in the H3K36 context (corresponding to −4 to +4 positions) (Fig. 1a) were substituted by all other proteinogenic amino acids, except for tryptophan and cysteine. Peptide arrays were incubated in methylation buffer with PRDM9 using radioactively labeled AdoMet as cofactor and the transfer of radioactivity analyzed by autoradiography (Fig. 1d) followed by quantitative analysis. Each experiment was performed in duplicates, which were normalized and averaged (Fig. 1d and Supplementary Fig. 2a). Analysis of the peptide spot specific error margins revealed very good reproducibility, with only one peptide with an error >10% in the H3K4 array, and only 3 peptides with errors >20% in the case of the H3K36 array (Supplementary Fig. 2b). The relative methylation activities on all peptides are shown in Fig. 2. In both cases, some mutant peptides showed higher methylation than the original H3K4 or H3K36 sequences. Hence, the peptide interface of PRDM9 is not perfectly optimized for any of the two targets, but somewhere in between, which illustrates one principle of the double recognition of two sequences by PRDM9. For further visualization purposes, the discrimination factor for each amino acid position was calculated (Fig. 2b), which describes the preference of PRDM9 for one specific amino acid residue at one site compared to all other residues at this place^24,25^.

A detailed analysis of these results revealed different interaction modes of PRDM9 with both peptides, explaining its ability for specific readout of two distinct amino acid sequences (Fig. 2). In case of the H3K4 peptide, sequence readout is established in the −3 to +2 range. At the −3 and −2 sites, strong and specific sequence readout is observed, in both cases favoring the natural amino acid A1 (−3 site) and R2 (−2 site). In combination with a preference for K at the −2 site, the data indicate that large basic residues are recognized here. At the −3 site, the most preferred residues A, T and V point towards a binding pocket for a methyl group in the amino acid side chain. In addition, specific interactions are observed at the −1, +1 and +2 sites. At these sites, however, the natural amino acids in the H3K4 sequence are only tolerated but other amino acids are clearly preferred. At the −1 site, large hydrophobic residues are preferred (V, I, L), while the natural T3 only shows moderate methylation. This implies that hydrophobic contacts are preferably formed with the enzyme at the −1 position. A strong preference of PRDM9 for I(−1) has also been observed in methylation studies of lysine-oriented peptide libraries indicating that these orthogonal approaches yield comparable results at sites with strong sequence recognition^26^. Similarly, at the +1 site the natural Q5 is tolerated, while T, V, and K are clearly favored. Of note, at the −1 and +1 positions, the preferred residues match with the H3K36 sequence (V35 and K37, see below). Finally, V is strongly preferred over the natural T6 at the +2 site suggesting that a larger hydrophobic binding pocket is available that cannot be filled by T.

In case of the H3K36 peptide, only weak readout is observed at the −3 position, where among other residues the natural G33 is preferred. At the −2 site, R is preferred as seen in the H3K4 peptide, but also the natural G34 which is not preferred in the H3K4 peptide, indicating that in the context of G33, G at the −2 site is preferred as well. This result suggests that two favorable conformations are possible for this part of the peptide, one including interaction with an R at the −2 site, and one based on the presence of G33 and G34. At the −1 site, I is strongly preferred in the H3K36 context, but the natural V35 is the next best residue. V and I are also preferred at the +1 site, indicative of a large hydrophobic binding site, but the natural K37 is the next preferred residue here. Hence, at these two places, the residues in the H3K36 peptide are clearly preferred over the ones in the H3K4 context. Finally, P38 is favored at the +2 site, which is interesting, because P is not among the favored residues at the +2 site in the context of the H3K4 peptide. This observation agrees with the previously made suggestion of H3P38 exploiting a hydrophobic cavity inside the catalytic site of PRDM9^14^. These data indicate that at this site the H3K36 peptide adopts a conformation that allows incorporation of P38 which is not accessible in the context of the H3K4 sequence.

Based on the H3K4 and H3K36 specificity profiles one can summarize the principles of dual peptide readout by PRDM9 as follows:

- The peptide interface of PRDM9 is not perfectly optimized for any of the two subtrates.
- In the N-terminal part of the peptide, there is strong and specific readout for A1 and R2 of the H3K4 peptide, possibly also including the N-terminal amino group. This provides binding and specific readout for the H3K4 peptide.
- In this region, the H3K36 peptide carries G33 and G34, which do not form interactions but adopt a H3K36 peptide specific conformation that prohibits the readout of A and R residues at these sites.
- In the central part of the peptides, from the −1 to the +2 site, the H3K4 residues are tolerated, but in general H3K36 residues are highly preferred. Hence, in this part of the peptide the specific binding and recognition of the H3K36 peptide is mediated.
- In the H3K36 sequence context (including the presence of K37), a specific conformation is available at the C-terminal part of the peptide allowing to bind P38 with high preference, although a P is not tolerated at this site in the H3K4 sequence context.

### Structural and molecular dynamics analysis of the H3K4 and H3K36 peptide recognition

**Fig. 3:**
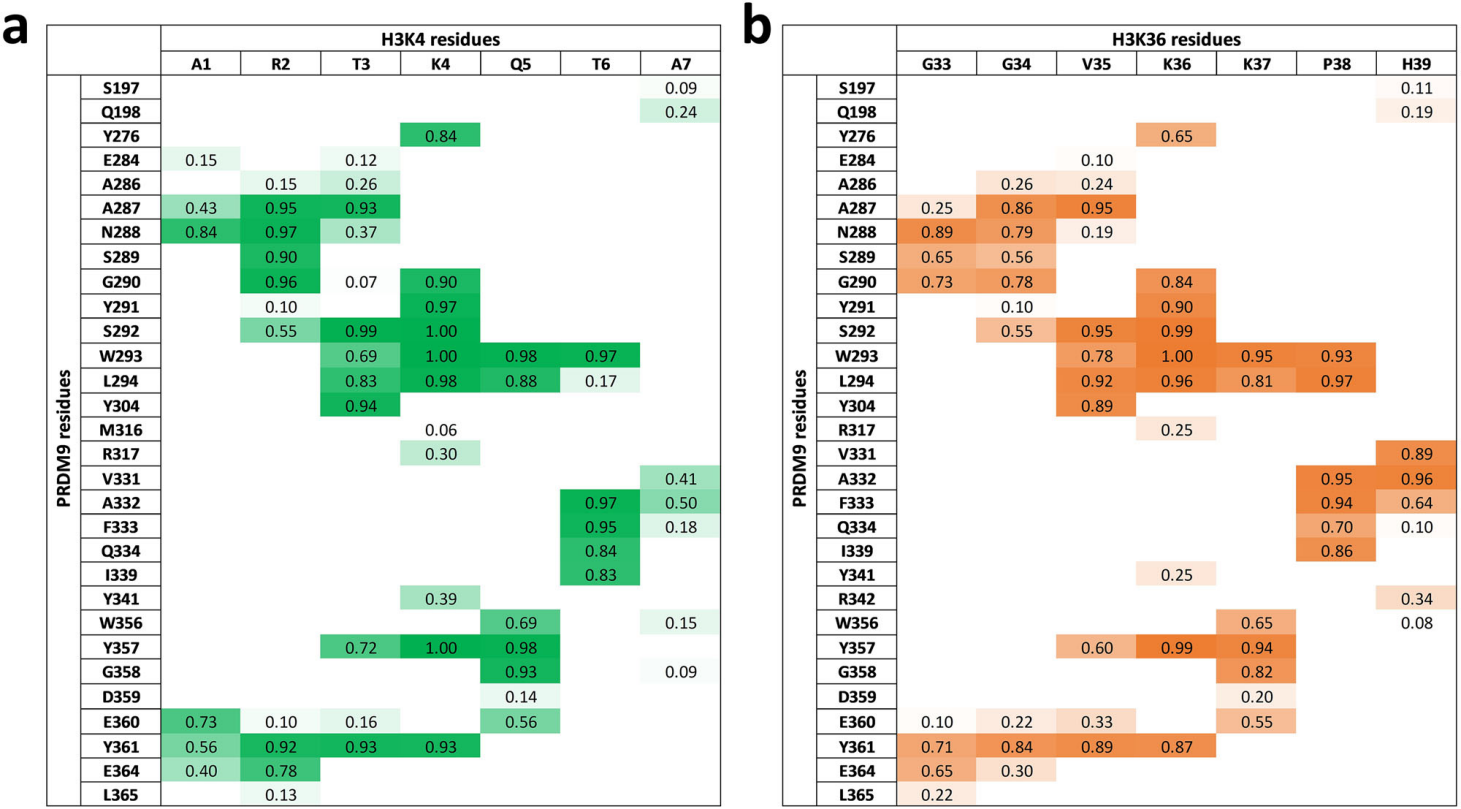
Contact maps of the H3K4 and H3K36 peptide side-chains with PRDM9 residues determined in the MD simulations. **a** Contact map of PRDM9 with bound H3K4 (1–7) peptide. **b** Contact map of PRDM9 with bound H3K36 (33−39) peptide. The values indicate the proportion of time in which certain contacts were established during the simulation. Contacts were considered as formed if the distance of a pair of heteroatoms from the side chain of the peptide and a PRDM9 residue was below 4.5 Å. In case of H3K36 G33 and G34, contacts were determined for all atoms.

**Fig. 4:**
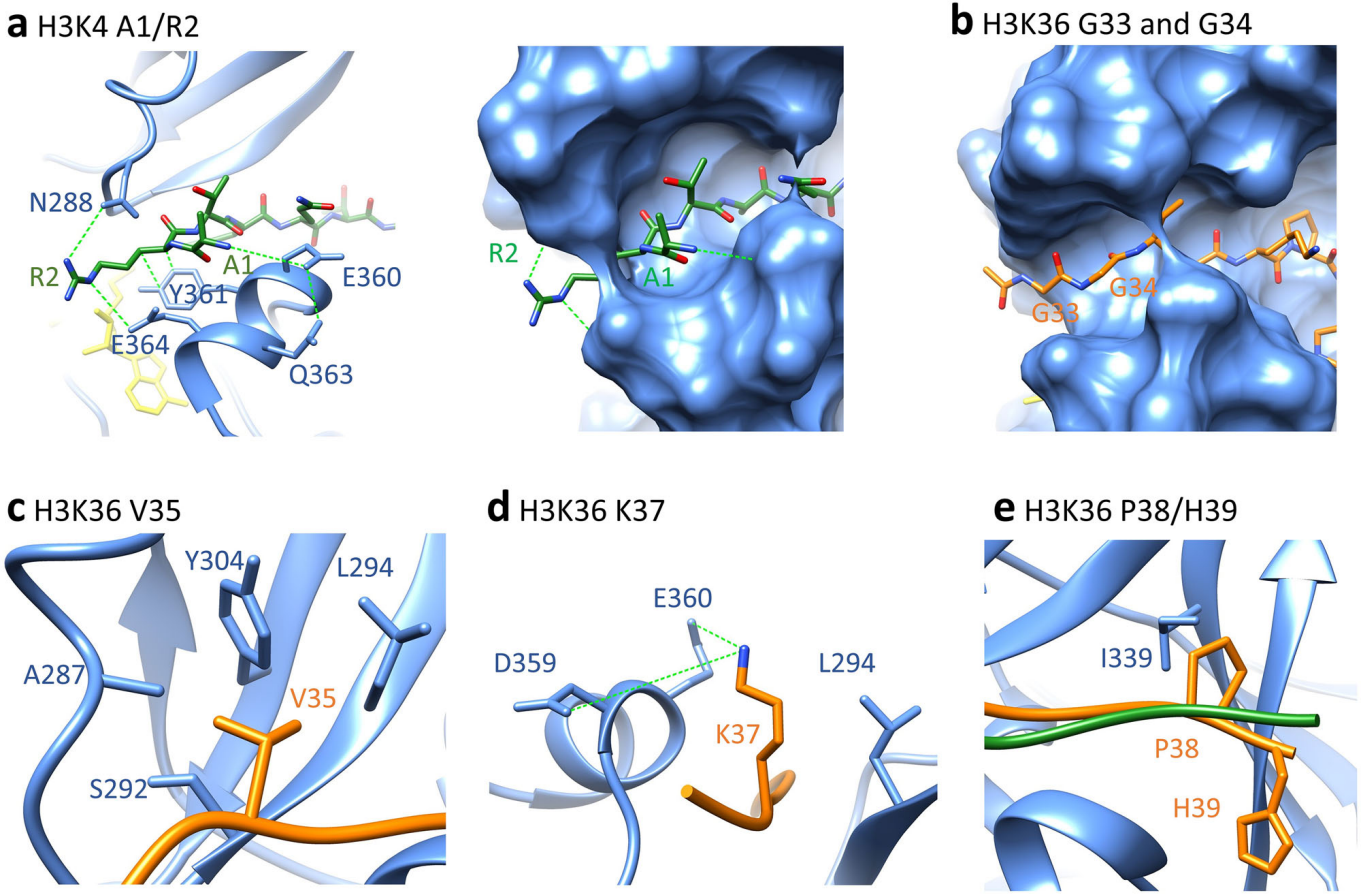
Representative conformations of PRDM9 SET domain in complex with H3K4 (1–7) or H3K36 (33−39) peptides observed in the MD simulations. The PRDM9 SET domain is colored in blue, the H3K4 peptide in green, and the H3K36 peptide in orange. Interactions are indicated by dotted green lines. **a** N-terminal positioning of the H3K4 peptide into the PRDM9 binding pocket with particular focus on the A1 and R2 recognition. **b** The corresponding positioning of the N-terminus of the H3K36 peptide following the trace of the R2 side chain in the H3K4 peptide. **c** Hydrophobic interaction of V35 in H3K36 with PRDM9 A287, S292, L294 and Y304 residues. **d** Contacts formed at position +1 between K37 of the H3K36 peptide and D359, E360 and L294 of the enzyme. **e** Conformation of the H3K4 and H3K36 peptide position +2 and +3 and interaction of H3K36 P38 with PRDM9 L294 and I339. All images were prepared with Chimera 1.18^56^.

To investigate the structural basis of the dual sequence readout of PRDM9, we used the available PRDM9 structure with bound H3K4 peptide (PDB 4C1Q^20^). To model PRDM9 complexed with H3K36, we used the existing SETD2 structure in complex with a H3K36 (29–43) peptide (PDB 5V21^27^), superimposed the PRMD9 and SETD2 enzymes, and placed the H3K36 peptide into the PRDM9 structure (all starting structures for MD simulations are available at 10.18419/darus-4567). Based on the structures and our specificity analyses, A1-A7 and G33-H39 peptides were used in the MD simulations as H3K4 and H3K36 specific substrates. The carboxy-terminal ends of both peptides were methylated to avoid the negative charge of the free acid that does not exist in a continuous protein chain. The H3K4 peptide was used with a free, positively charged amino group at A1, while the H3K36 peptide was used with an acetylated N-terminus to mimic the next peptide bond. After addition of ions and solvent, 21 MD simulations à 100 ns were performed for each complex (with independent equilibration after 3 cycles) (Supplementary Fig. 3) and frames were recorded every 20 ps. To investigate the molecular mechanisms behind the recognition of the H3K4 and H3K36 sequences, maps of the contacts between the side chains of each of the peptides and PRDM9 during the simulation were prepared using contact map explorer (Swenson, D. E. H. & Roet, S.: Contact Map Explorer. https://github.com/dwhswenson/contact_map). Contacts were considered as formed if the distance of a pair of heteroatoms from the peptide amino acid side chains and a PRDM9 residue was below 4.5 Å. In case of H3K36 G33 and G34, contacts were determined for all atoms. The fraction of time in which a contact was established during the simulation was measured and used to create contact profiles (Fig. 3). In addition, representative conformations of the complexes were extracted and used for visualization of the data (Fig. 4).

#### Peptide interaction at the −3 and −2 site

At the N-terminal end, the free amino group of H3K4-A1 interacts with E360. In this conformation, the peptide chain could not be continued as it would clash into a loop of PRDM9 formed by D359-E364. H3K4-R2 is contacted with H-bonds by N288 and E364 as well as by hydrophobic interactions from Y361 (Fig. 4a). Moreover, R2 is inserted into a narrow channel, which altogether explains the preference for R and K at the −2 site of H3K4 (Fig. 4a). In the H3K36 peptide, G33 and G34 are located in the channel occupied by the side-chain of R2 in the H3K4 peptide (Fig. 4b). The H3K36 specific conformation depends on the presence of G33 and G34 at the −2 and −3 sites, which are the smallest and most flexible of all amino acids, explaining why G is preferred at the −2 site, if G33 occupies the −3 site. This conformation provides an open path to the surface of PRDM9 allowing the bound peptide to be continued, hence it is the only available conformation for H3K36 methylation.

#### Peptide interaction at the −1 site

At the −1 site, a relatively large hydrophobic pocket is formed by the side chains of A287, L294 and Y304 which explains the preference for I, V and L at this place and ideally accomodates H3K36 V35 (Fig. 4c). At the bottom of this pocket, S292 provides a H-bond option, explaining why T3 in the H3K4 peptide can also bind. However, the single methyl group of T3 is not large enough to fill the hydrophobic pocket explaining the lower preference for this residue.

#### Peptide interaction at the +1 site

At the +1 position, the contact pattern of H3K4 Q5 and H3K36 K37 does not differ much. The +1 site is flanked by the side chain of L294 explaining the preference for V and I, but the alkane chain of K37 can also interact with L294 (Fig. 4d). In addition, D359 and E360 can engage in electrostatic contacts with K37, but they are too far away to generate an H-bond with Q5, which allows to rationalize the preference for K37 at this site, combined with a lack of preference for Q5.

#### Peptide interaction at the +2 site

The +2 site is in close contact with I339 leading to a pronounced preference for V in both peptide contexts (Fig. 4e). However, H3K36 P38 also showed a good fit, while H3K4 T6 is not favored. This effect is accompanied by L294 forming a strong contact to H3K36-P38, while it only weakly interacts with H3K4-T6, which could be related to the positioning of L294 by the H3K36-K37 alkane chain, that cannot be provided by H3K4-Q5. P38 causes a kink of the peptide chain, which leads to stronger contacts of H3K36-H39 when compared with H3K4-A7, along with an interaction formed between H39 with R342. This interaction, present only in the H3K36 contact profile, may also explain the novel preference for E at the +3 site which is specific for the H3K36 peptide sequence.

### H3K4 and H3K36 methylation activity with soluble peptides

**Fig. 5:**
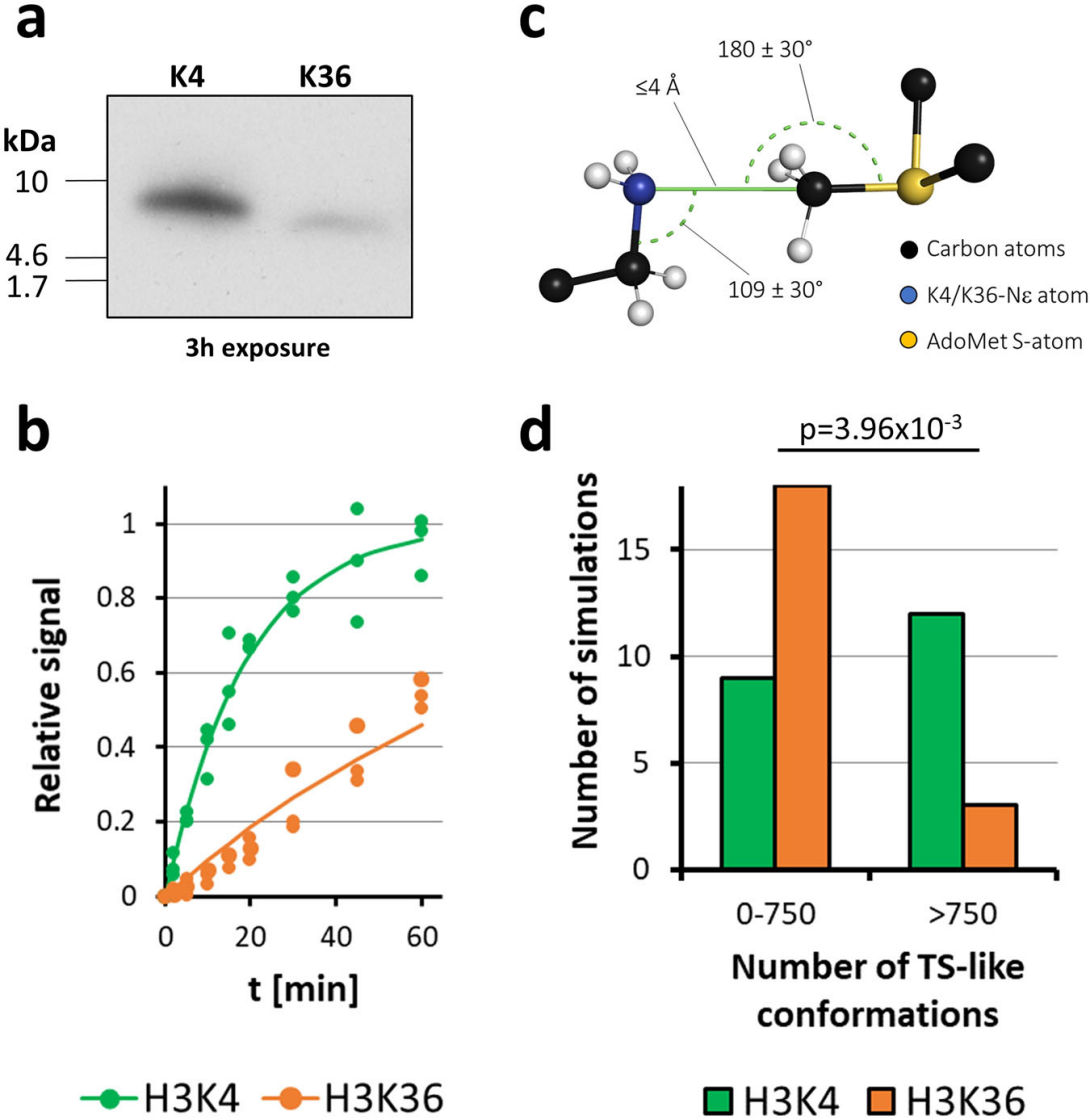
Methylation activity of PRDM9 on H3K4 and H3K36 peptides observed in experiments and MD simulations. **a** Exemplary autoradiographic image of soluble H3K4 (1−19) and H3K36 (26–44) peptides methylated by PRDM9 (195–415) and separated by Tricine-SDS-PAGE. **b** Kinetics of H3K4 and H3K36 methylation by PRDM9 (195–415). Data were fitted to an exponential reaction progress curve yielding a ratio of 5.2 for the initial methylation rates of H3K4/H3K36. Data points show results of three independent experiments with each substrate, lines show the combined fit of the data. Exemplary gel images are shown in Supplementary Fig. 4a. **c** Representation of the characteristic distances and angles between the Nε-atom of the target lysine, the C-atom of the AdoMet methyl group, and the S-atom of AdoMet for a PKMT TS-like conformation. **d** Distribution of the number of TS-like conformations observed in each of the 21 individual MD simulations with the H3K4 and H3K36 substrates. The p-value was determined by Fisher’s exact test.

Following the investigation of the specificity of PRDM9 for its two target sequences, we aimed to determine the relative activities of WT PRDM9 with both substrates. Peptide SPOT array methylation assays are not suitable to compare the activity of several mutants, because they cannot be processed in parallel. We, therefore, resorted to methylation assays using purified peptides. Two HPLC purified substrate peptides H3K4 (1–19) and H3K36 (26–44) were purchased and their methylation determined using peptide and PRDM9 concentrations of 2.5 µM and 50 nM, respectively, in methylation buffer supplemented with radioactively labeled AdoMet. Afterward, the samples were separated on Tricine gels and the transfer of radioactivity to the peptides was determined by autoradiography and analyzed quantitatively (Fig. 5a). As a first experiment, we compared the relative methylation rates of the H3K4 and H3K36 peptides and found that the H3K4 peptide was methylated about 5.2-times faster (Fig. 5b and Supplementary Fig. 4a) which is consistent with previously reported in vitro methylation activities of human and murine PRDM9^14,15^. Additional experiments demonstrated that this ratio is stable over a range of AdoMet concentrations indicating that AdoMet concentrations do not affect the peptide substrate preference (Supplementary Fig. 4b).

MD simulations do not allow breaking and formation of bonds. Hence, they cannot be used for direct analysis of reaction rates. However, our previous work has shown that in the case of PKMTs adoption of conformations that resemble the known S_N_2 transition state geometry of the methyl group transfer in PKMT catalyzed reactions (TS-like conformations) (Fig. 5c) are a suitable criterion to estimate the corresponding catalytic activities^22,28–30^. We, therefore, inspected how often TS-like conformations occurred in the simulations with the H3K4 and H3K36 peptide, showing that simulations with many active conformations were much more abundant with the H3K4 peptide complex than with the H3K36 peptide complex where more simulations showed only few TS-like states (Fig. 5d). Binning the simulations into high and low frequency of TS-like conformations followed by statistical analysis based on Fisher’s exact test confirmed significance of this finding with a p-value of 3.96 × 10^−3^ (Fig. 5d). Quantitatively, hyperactive states were observed 4-times more frequently with the H3K4 substrate than with H3K36, which roughly fits with the biochemical result showing that the simulations can describe the underlying processes remarkably well.

### Investigation of PRDM9 residues forming enzyme-peptide contacts

**Fig. 6:**
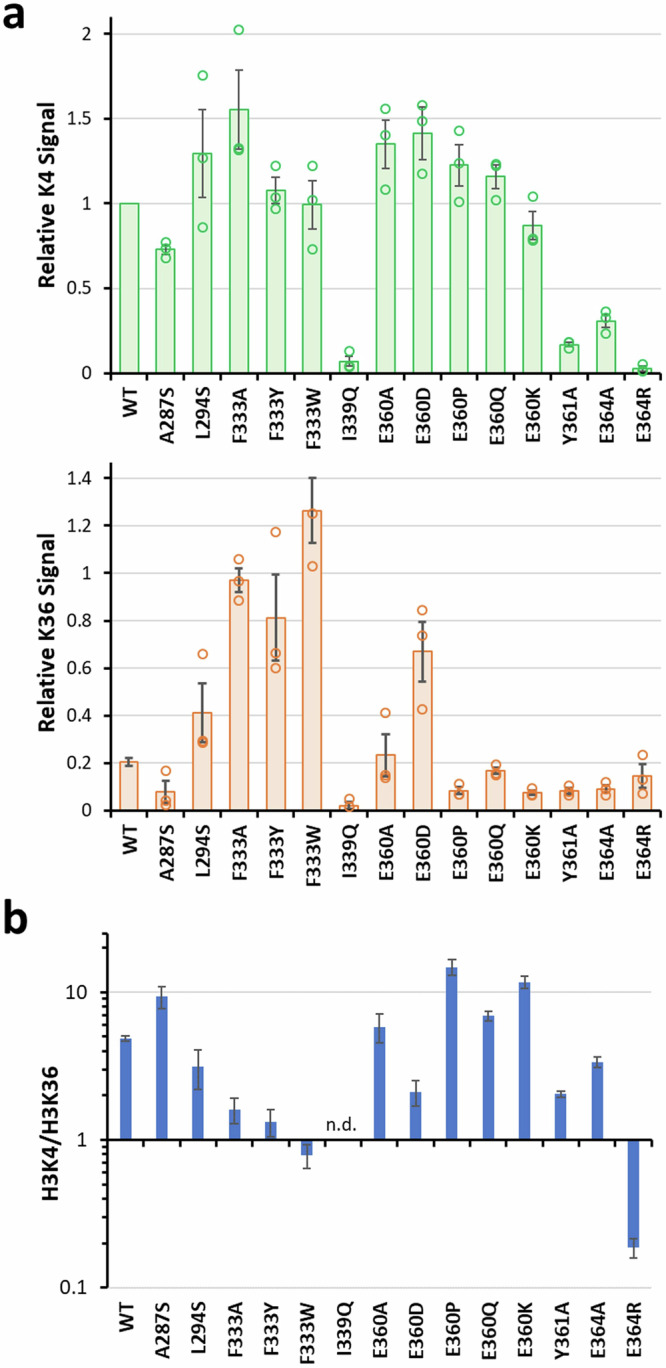
Relative methylation rates of the H3K4 and H3K36 peptides by WT and mutant PRDM9 enzymes. Analysis of the autoradiography signal from the soluble peptide H3K4 (1–19) and H3K36 (26–44) methylation by PRDM9 (195–415) WT and mutant enzymes. **a** Relative methylation signal intensities of H3K4 and H3K36 peptide bands normalized to the WT H3K4 methylation signal. The bars show the average of 3 independent experiments (21 in the case of the WT enzyme), the error bars display SEM. **b** Representation of the H3K4/H3K36 methylation signal ratio. The data were taken from (**a**), the errors were propagated from (**a**).

Next, we aimed to investigate the mechanism of peptide recognition by mutational analysis of residues that putatively are involved in peptide binding. For this, we focused on the PRDM9-peptide interactions potentially mediating specific readout and analyzed their effects on the specific activity on H3K4 and H3K36 peptide substrates. We selected 7 PRDM9 residues forming contacts to the H3K4 or H3K36 peptides (A287, L294, F333, I339, E360, Y361, and E364) and investigated altogether 14 PRDM9 mutants. The mutants were generated, purified, and their concentrations carefully adjusted using the purified WT protein as reference (Supplementary Fig. 5). Next, the catalytic activities of all mutants were determined with the H3K4 and H3K36 peptides in 3 experimental repeats (Supplementary Fig. 6). The data were analyzed and compared with the K4/K36 preferences of WT PRDM9 (Fig. 6).

#### Residues with −1 site interaction

At the substrate position −1, the side chain of A287 forms a hydrophobic contact to the H3K4-T3 and H3K36-V35 and constraints the size of peptide binding pocket (Fig. 4c). The specificity profile revealed a preference for larger aliphatic amino acids at this position in both sequence contexts indicating that V35 in the H3K36 substrate is favored over the T3 in the H3K4 substrate. To challenge this hypothesis, we mutated the A287 to S, which could still form a hydrogen bond to T3 but no longer engage in hydrophobic interactions. Using this mutant, the methylation of the H3K4 and H3K36 peptides was measured and compared with WT PRDM9. We found that the total methylation activity of A287S was reduced; however, the effect was more pronounced with H3K36 such that the preference for the H3K4 peptide was increased (Fig. 6), which is in agreement with our hypothesis.

#### Residues with −2 site interaction

The R2 residue at the substrate position −2 of the H3K4 peptide potentially contacts Y361 and E364 by electrostatic or hydrophobic interactions (Fig. 4a). Y361 was previously reported to aid in the positioning of target lysine into the active site by being part of the substrate lysine binding channel^20^. Overall, these contacts were expected to favor H3K4 methylation, because the H3K36 peptide carries G34 at this site, which is not able to engage in similar contacts. By creating single mutants of Y361 and E364 to alanine, we aimed to disrupt these H3K4 preferring interactions. Moreover, Y361 was reported to aid in the positioning of target lysine into the active site by being part of the substrate lysine binding channel^20^. Indeed, both mutants showed strongly reduced overall activities. However, in agreement with our hypothesis, the decline in activity was less pronounced with the H3K36 peptide in both cases leading to a reduction in the preferences for H3K4 methylation (Fig. 6). To further enhance the effects of the PRDM9 specificity engineering, E364 was mutated to R, which indeed abolished H3K4 methylation completely leading to a total reversal of the preference of the E364R mutant towards methylation of the H3K36 peptide, further emphasizing the importance of this residue for H3K4 target recognition.

#### Residues with +1 site interaction

In the MD simulations, the H3K36 +1 site residue (K37) is oriented towards E360 (Fig. 4d). However, the distance of E360 is too large for an H-bond to form with Q5 at the corresponding position in the H3K4 peptide, which was expected to support methylation of the H3K36. We generated and tested the E360P and E360K mutants, which were studied already previously^21,31,32^, together with E360A, E360Q, and E360D to determine the role of this residue in peptide recognition in detail. Most of the E360 mutants had no crucial effect on H3K4 methylation except a slight reduction with the E360K mutant (Fig. 6a) which is in line with the lack of interaction of this residue with the H3K4 peptide. The mutations of E360 to alanine or glutamine also did not affect H3K36 methylation and, consequently, these mutants did not show noticeable changes in methylation preferences (Fig. 6). In contrast, the E360P and E360K mutants were found to methylate H3K36 with a strongly reduced rate, increasing their preferences for H3K4 methylation which is in agreement with previous findings^21,32^. The proline at position 360 likely adds rigidity to the C-terminal helix of PRDM9, which might hinder the H3K36 peptide from productive binding, while the lysine at position 360 presumably disfavors the H3K37 interaction because of charge repulsion. An opposite shift in specificity was observed with the E360D mutant, which showed elevated activity on the H3K36 substrate presumably by allowing an even better interaction with K37 while further weakening the residual contact with Q5 due to its shorter side chain when compared with the original E. As a consequence, E360D showed a reduced K4/K36 activity ratio.

Since the specificity analysis indicated, that the most preferred substitutions on position +1 are either valine or isoleucine, the contact of residues at this site with L294 is likely to be involved in peptide recognition as well. After mutating L294 to serine, the methylation of the H3K36 peptide was increased while H3K4 methylation was not altered (Fig. 6a), showing a similar shift of the preference towards H3K36 as the E360D mutant (Fig. 6b). One may speculate that the exchange of the hydrophobic L294 with the shorter polar S reliefs the tight binding of this residue between K37 and P38 in the H3K36 peptide. This could increase the flexibility of K37 and allowing it to interact better with D359 and E360 and enable P38 to position in a more relaxed conformation.

#### Residues with +2 site interaction

Regarding the +2 position of H3K4 and H3K36, it was previously assumed that H3K36-P38 binds to a hydrophobic cavity, which is occupied by H3K4-T6 in the H3K4 context^14^. Mutation of the I339 which is part of this hydrophobic binding site to a Q, however, showed an almost complete loss of methylation of both H3K4 and H3K36 which is in agreement with our MD simulations showing that I339 interacts with H3K36-P38, but also H3K4-T6. However, it was anticipated that Q339 might be able to engage in a H-bond with H3K4-T6, which apparently either is not the case, or this leads to stabilization of an inactive conformation of the PRDM9-H3K4 peptide complex.

Moreover, in the crystal structure of PRMD9 with bound H3K4 pepitde (pdb 4C1Q,^20^), H3K4-T6 interacts with the backbone of F333, which is part of a β-sheet and stabilized by stacking interactions with F340 and R342 (Supplementary Fig. 7). Based on this, we speculated that F333 might constraint the space in the peptide binding pocket at the +2 site, perhaps contributing to the relative disfavor for H3K36 which contains the larger P38 and also introduces a bent in the peptide backbone. We aimed to weaken the docking of F333 by exchanging it to alanine, tryptophan, or tyrosine, expecting that this should provide more flexibility in the peptide binding pocked. Indeed, a strong increase in H3K36 methylation could be observed with all three mutants without a noticeable effect on H3K4 methylation (Fig. 6). Presumably, the more flexible interface allows a more relaxed positioning of P38, similarly as seen with the L294S mutation. This leads to an increase in preference for H3K36 peptide methylation compared to H3K4 peptide methylation. However, in the biological context it is possible that the close interaction of F333 and P38 is necessary for specificity of PRDM9 by counterselection against other residues at the +2 site of the peptide. Then, the mutants with higher H3K36 activities generated here might not be selected in nature, due to their potential disadvantage of a reduced specificity leading to methylation of additional non-histone substrates.

#### Evolutionary implications

There are 52 reviewed, human proteins listed at InterPro (https://www.ebi.ac.uk/interpro/) that contain a SET or PRDM domain. These proteins are the outcome of divergent evolution^22,33–35^ and many of them have been validated as PKMTs with a diverse set of histone and non-histone substrates. Therefore, PKMTs have changed their substrate specificity several times during divergent evolution. We observed that PRDM9 methylates the H3K4 peptide about 5-fold faster than the H3K36 peptide. However, PRDM9 single mutants showed a wide variety of K4/K36 preferences, some of them had elevated preference for H3K4, others methylated both substrates equally, and some mutants even displayed a preference for the H3K36 substrate. In these cases, our mutational data document a change of the substrate specificity of PRDM9 by an exchange of single amino acids residues illustrating potential pathways in the divergent evolution of SET-domain and PRDM-domain PKMTs leading to diverse substrate specificities.

## Discussion

The PRDM9 protein lysine methyltransferase specifically interacts with H3K4 and H3K36 peptides despite the fact that their amino acid sequences are almost completely distinct (no residue besides the target lysine and an R at the +4 position is identical in both peptides). By combining biochemical and modeling techniques, we show here that this exceptional dual substrate specificity is based on a bipartite peptide recognition cleft, comprising one peripheral binding site specific for the H3K4 peptide but tolerating H3K36, and one central binding site with the opposite preferences.

The N-terminal part of the H3K4 peptide and R2 are bound by a network of PRDM9 residues in a bent conformation that sterically excludes the continuation of the peptide chain in N-terminal direction. The H3K36 peptide contains G33 and G34 in this part allowing the peptide chain to occupy the binding path of the R2 side chain in the H3K4 complex. Because of this, a continuous peptide can be bound in the H3K36 binding mode. Conversely, in the central part of the substrate binding pocket, hydrophobic residues as those found in H3K36 are highly preferred, enabling an accurate readout and efficient methylation of the H3K36 sequence. Still, the residues found in H3K4 in this region are tolerated which in combination with the strong recognition of the N-terminal part of H3K4 in the peripheral region of the binding site allows to specifically methylate the H3K4 substrate as well. Peptide methylation and modeling data revealed an about 5-fold overall preference for methylation of H3K4 (H3K4 > H3K36).

It is one limitation of the current study that all experiments were conducted in vitro using peptide substrates and purified PRDM9 because substrate preferences may be altered in the context of chromatin and in the presence of additional interacting proteins. However, our study provides compelling insights into the biochemical mechanisms governing the dual substrate specificity of PRDM9 and, based on our data, site-directed mutagenesis of residues involved in PRDM9-peptide contacts allowed us to modulate the K4/K36 preferences strongly. In our protein engineering experiments, PRDM9 mutants were identified with elevated preference for H3K4 (H3K4 >> H3K36), with lost preference (H3K4 ≈ H3K36), and even with inverted preference (H3K36 > H3K4). One additional mutant showed very low activity I339Q mutant, but in this case misfolding of the purified protein cannot be excluded. These findings document that the remarkable substrate recognition by PRDM9 with dual specificity can be tuned towards the preference for one or the other peptide. By this, our data also illustrate potential evolutionary pathways to modulate and finally swap the sequence specificity of PKMTs by few amino acid exchanges in the enzyme, a process that happened several times in the divergent evolution of SET- and PRDM-domain PKMTs.

## Methods

### Cloning, expression, purification of PRDM9 WT and mutants

The PRDM9-pET28-MHL plasmid (195–415) was acquired from Addgene (Plasmid: #51328). All PRDM9 mutants were cloned by performing the site-directed mutagenesis method using the PRDM9-pET28-MHL plasmid as template and validated by Sanger sequencing (Microsynth Seqlab GmbH). For protein expression BL21-DE3 codon plus *E. coli* cells were transformed with the corresponding plasmids and cultured in LB media, supplemented with 25 µg/ml kanamycin, at 37 °C until an OD_600_ of 0.6–0.8 was reached. The expression was induced overnight at 20 °C by adding IPTG (500 mM). Cells were harvested by centrifugation at 4500 rpm for 25 min. The cell pellets were washed once with STE buffer (100 mM NaCl, 10 mM Tris-HCl pH 8.0, 1 mM EDTA) and centrifugated a second time at 4500 rpm for 25 min before they were stored at −20 °C. Protein purification was carried out at 10 °C by resuspending the cell pellets first in Sonication buffer (30 mM KPi pH 7.2, 0.5 M KCl, 0.2 mM DTT, 1 mM EDTA, 20 mM imidazole, 10% glycerol) supplemented with Protease Inhibitor Cocktail (to a final concentration of 749.3 μM AEBSF-HCl, 7.5 μM pepstatin A, 0.3 μM aprotinin, 37.5 μM bestatin, 11.4 μM E-64, 16.7 μM leupeptin). Cells were sonicated 15 times with 15 s impulse (4 cycles, 30% power) and 45 s off-time per repeat (Sonoplus UW2200, Bandline). The lysed cells were then centrifuged (18,000 rpm, 1.5 h) and the cleared lysate was loaded onto 500 µL of Ni-NTA agarose beads (QIAGEN) previously equilibrated to the Sonication buffer. Thereafter, the sample on then column was washed with 60 ml Sonication buffer and eluted with Elution buffer (30 mM KPi pH 7.2, 0.5 M KCl, 0.2 mM DTT, 1 mM EDTA, 220 mM imidazole, 10% glycerol) followed by a 2 h dialysis step in Dialysis buffer (20 mM HEPES pH 7.2, 0.2 M KCl, 0.2 mM DTT, 1 mM EDTA, 10% glycerol). The protein was stored in aliquots at −80 °C until use.

### Peptide SPOT array methylation

Peptide arrays were synthesized on a cellulose membrane using the SPOT synthesis method^36^ with a Multipep peptide synthesizer (CEM). Each peptide spot contains ~9 mmol of peptide (Multipep Reference Handbook, CEM). The successful synthesis of each peptide was confirmed by bromophenol blue staining^24,25^. Prior to methylation, the arrays were incubated in methylation-buffer (10 mM Tris/HCL pH 8.5, 10 mM DTT, and 0.01% Triton X) for 5 min at RT. The methylation reaction was carried out for 1 h at RT using the same methylation-buffer (10 mM Tris/HCL pH 8.5, 10 mM DTT, 0.01% Triton X) supplemented with 48 nM AdoMet (Perkin Elmer Inc., dissolved at dissolved at 0.7 µM in 10 mM sulfuric acid) and 100 nM PRDM9. Thereafter, the arrays were washed 5 times each for 5 min with wash-buffer (100 mM NH_4_HCO_3_ and 1% SDS) and incubated once for 5 min in ENLIGHTING Rapid Autoradiography Enhancer (Perkin Elmer Inc.). The detection of methylated substrates was performed by autoradiography after different times of exposure. The signal spot intensity was analyzed with the Phoretix Array software (Totallab life science analysis). Each spot intensity was normalized based on the total intensity maximum and minimum of the corresponding array before calculating the average and the mean absolute error between both replicates.

### Methylation of soluble peptides

Soluble H3 1–19 (H-ARTKQTARKSTGGKAPRKQ-NH_2_) and H3 26–44 (Ac-RKSAPATGGVKKPHRYRPG-NH_2_) peptides were ordered from Shanghai Royobiotech Co. Ltd. in HPLC purified form (purity >95%). Their identity was validated by mass spectroscopy. Peptide methylation was carried out in a total volume of 20 µl (2.5 µM of the corresponding peptide, 50 nM enzyme, 20 mM Tris HCl pH 8.5, 20 mM DTT, and 0.02% Triton X) by the addition of 34 nM radioactively labeled AdoMet (Perkin Elmer Inc., dissolved at 25 µM in 10 mM sulfuric acid) for 30 min at RT. The methylation reactions were stopped by the addition of 5x SDS-PAGE loading buffer and heating at 95 °C for 10 min. Reaction samples were separated by Tricine-SDS-PAGE and the methylation was detected by autoradiography. ImageJ was used to measure the band intensity, which was normalized to the H3K4 wild type signal.

Methylation kinetics of H3K4 and H3K36 were conducted the same, but samples were incubated variable times before stopping the methylation reaction. Methylation reactions of H3K4 and H3K36 were analyzed side-by-side on one x-ray film to allow direct comparison of both signals. Signals of individual experiments were normalized to the H3K4 signals for quantitative analysis. Methylation intensities were fitted to mono-exponential reaction progress curves using the same A and B values for the fits of the H3K4 and H3K36 methylation signals:

$${{\rm{Signal}}}({{\rm{H}}}3{{\rm{K}}}4)={{\rm{A}}}+{{\rm{B}}}\; (1-\exp ({-{{\rm{k}}}}_{{{\rm{H}}}3{{\rm{K}}}4}{{\rm{t}}}))$$

$${{\rm{Signal}}}({{\rm{H}}}3{{\rm{K}}}36)={{\rm{A}}}+{{\rm{B}}}\; (1-\exp ({-{{\rm{k}}}}_{{{\rm{H}}}3{{\rm{K}}}36}{{\rm{t}}}))$$

With: kH3K4, rate of H3K4 methylation; kH3K36, rate of HK3K36 methylation.

Afterward, the k_H3K4_/k_H3K36_ value indicates the ratio of initial methylation rates of both substrates. Another set of control reactions was conducted with addition of unlabeled AdoMet (Sigma) of up to 1 µM.

### MD Simulation of the PRDM9-peptide complexes and trajectory analysis

All molecular dynamics (MD) simulations were performed in OpenMM 7.5.1^37,38^ utilizing the NVIDIA CUDA^39^ GPU platform. The systems were parameterized using the General Amber force field (GAFF) and AMBER 14 all-atom force field^40,41^. The non-bonded interactions were treated with a cut-off at 10 Å. Additionally, the Particle Mesh Ewald method^42^ was used to compute long-range Coulomb interactions with a 10 Å nonbonded cut-off for the direct space interactions. Energy minimization of the system was performed until a 10 kJ/mole tolerance energy was reached. Simulations were run using a 2 fs integration time step. The Langevin integrator^43^ was used to maintain the system temperature at 300 K with a friction coefficient of 1 ps^−1^. The initial velocities were assigned randomly to each atom using a Maxwell–Boltzmann distribution at 300 K. A cubic water box with a 10 Å padding to the nearest solute atom was filled by water molecules using the tip4p-Ew model^44^. An ionic strength of 0.1 M NaCl was applied, by adding the corresponding number of Na^+^ and Cl^−^ ions (specified later). Protonation states, equilibration protocols and other specifications for the individual system setups are described below. Production runs were performed under periodic boundary conditions, and trajectories were written every 10,000 steps (20 ps).

For the MD simulations of PRDM9 complexed with each peptide, the structure of human PRDM9 (positions A195–I367) was modeled based on the crystal structure of PRDM9 in complex with the seven amino acid long H3 peptide with K4 as the target lysine, called H3K4 (residues A1-A7, PDB 4C1Q, Chain A)^20^. Since no structure of PRDM9 complexed with the H3 peptide with K36 as the target lysine is available, a crystal structure of SETD2 complexed with 15 amino acid long H3K36 peptide (A29-P43) was used as a template (PDB 5V21)^27^. PRDM9 and SETD2 were superposed using PyMOL (2.4.1)^45^ and H3K36 transferred to the PRDM9 structure (all starting structures for MD simulations are available at 10.18419/darus-4567).

To prevent unnatural charged interactions of the artificial C-terminus for H3K4 and N- and C-terminus for H3K36, both peptides were modified. Both C-terminal ends were manually methylated to avoid the negatively charged terminal carboxyl group. The N-terminus of H3K36 was manually acetylated to avoid a positively charged amino group. The N-terminus of H3K4 was used in unmodified form since this is the natural state the enzyme is interacting with. The K4 and K36 target residues were manually deprotonated as required for the S_N_2 mechanism^22,46,47^. AdoMet was modeled based on the coordinates of SAH in PDB 4C1Q and parametrized using ANTECHAMBER from AmberTools (18.0)^48^. The Zn^2+^ ion was modeled using the cationic dummy atom method^49–51^. Cysteines 205, 208, 216 were treated as unprotonated to ensure proper Zn^2+^ binding^52^. The protein charge was neutralized and an ionic strength of 0.1 M NaCl was applied. This was facilitated by adding 27 Na^+^ and 15 Cl^−^ ions for the system with the complexed H3K4 peptide and 28 Na^+^ and 15 Cl^−^ for the system with the H3K36 peptide complexed with PRDM9. Further information about the simulated systems are provided in Supplementary Table 1.

To equilibrate the solvent, a 5 ns pressure coupled equilibration with Monte Carlo barostat^53^ was performed at a pressure of 1 atm. Initially, the Cα atoms of PRDM9 were restrained with a force constant of 50 kJ mol^−1^ Å^−2^, and the peptide and AdoMet atoms were restrained with a force constant of 2 kJ mol^−1^ Å^−2^. The restraints were removed stepwise, starting with a 5 ns equilibration with restraints only on the peptide and AdoMet, followed by 5 ns equilibration with no restraints. Subsequently, 3 production runs were conducted, 100 ns each. This was done 7 times for each peptide, leading to 21 replicates (2.1 µs total simulation time each) (Supplementary Fig. 3). Conducting a fresh equilibration every 3 replicates was done to minimize potential effects of the equilibration phase on the production runs.

In order to define criteria describing a catalytically competent conformation, the following geometric requirements for a PKMT transition state (TS)-like conformation were derived from the known S_N_2 geometry of methyl group transfer reaction^22,28^ (Fig. 5c).

1. The distance between the lysine Nε and AdoMet methyl group C-atom is <4 Å.
2. The angle between the lysine Nε, the lysine Cδ bond, and the virtual bond between lysine Nε and the AdoMet methyl group C-atom is in a range of 109° ± 30°.
3. The angle between the lysine Nε, the AdoMet methyl group C-atom and AdoMet S-atom bonds is in a range of 180° ± 30°.

Data analysis was performed utilizing MDTraj (1.9.4)^54^ to calculate the distances and angles necessary for the geometric criteria of an S_N_2 TS-like conformation. The contact map analysis was performed with contact-map explorer (0.7.1)^55^. For the contact maps a cut-off of 4.5 Å was used for the analysis. A contact was counted if at least one heteroatom of a residue was in a 4.5 Å^3^ sphere surrounding one heteroatom from another residue excluding neighboring residues. For peptide residues the side-chains were used, except in case of G, where all atoms were considered. All structures were visualized using Chimera 1.18^56^.

### Statistics and reproducibility

The number of independent experimental repeats is indicated for each experiment. Standard deviations were determined with MS Excel. P-values were determined using Fisher’s exact test. Sample sizes are indicated in the text and Figure legends.

### Reporting summary

Further information on research design is available in the Nature Portfolio Reporting Summary linked to this article.

## Supplementary information

Supplementary Information

Description of Additional Supplementary Files

Supplementary Data 1

Reporting Summary

## Data Availability

All biochemical data generated or analyzed during this study are included in the published article and its supplementary files. Uncropped images of the Figures and Supplementary Figures are provided in Supplementary Fig. 8. The source data behind the graphs in the paper can be found in Supplementary Data 1. All PDB files and MD simulation protocols used in this study are deposited at DaRUS (10.18419/darus-4567), including modeled structures of PRDM9 bound to different peptides, starting structures of the MD runs, a movie of the MD run, source data of the results of the MD analysis, MD simulations codes and analysis scripts.

## Code availability

MD simulation codes and analysis scripts are provided on DaRUS (10.18419/darus-4567).
