# Supplementary material for: Investigation and design of the dual specificity of the PRDM9 protein lysine methyltransferase
Source: Commun Biol. 2025 May 29;8:823. doi: 10.1038/s42003-025-08207-4 (PMC12122940; doi:10.1038/s42003-025-08207-4)
Supplement: Supplementary file 1 — Supplementary Information [file 42003_2025_8207_MOESM1_ESM.pdf]

Investigation and design of the dual specificity of the PRDM9 protein lysine methyltransferase

Dimitri Graf, Philipp Schnee, Jürgen Pleiss, Sara Weirich, Albert Jeltsch

Supplementary Tables

Supplementary Table 1: Additional information about the MD simulated systems.

Supplementary Figures

Supplementary Figure 1: Comparison of PRDM9 with H3K4 and H3K36 PKMTs.

Supplementary Figure 2: Additional information related to Figure 1d and Figure 2.

Supplementary Figure 3: Schematic picture of the setup of the MD simulations.

Supplementary Figure 4: Additional information related to Figure 5.

Supplementary Figure 5: Coomassie Brilliant Blue stained SDS gel electrophoresis of purified PRDM9 (195-415) WT and mutants.

Supplementary Figure 6: H3K4 and H3K36 methylation activity of WT and mutant PRDM9 enzymes.

Supplementary Figure 7: Image showing the interaction between the +2 position of the H3K4 peptide and PRDM9 (195-415) derived from the crystal structure (pdb 4C1Q).

Supplementary Figure 8: Uncropped images of the Figures and Supplementary Figures

Supplementary Table

Supplementary Table 1: Additional information about the MD simulated systems.

	**PRDM9-H3K4-SAM**	**PRDM9-H3K36-SAM**
Number of MD simulation production runs	21	21
Simulation box dimensions	63 Å	63 Å
Total number of atoms	36439	36715
Total number of water molecules	8432	8502
Salt concentration	0.1 M	0.1 M
Lipid composition (number of molecules and type)	Not applicable	Not applicable

Supplementary Figures

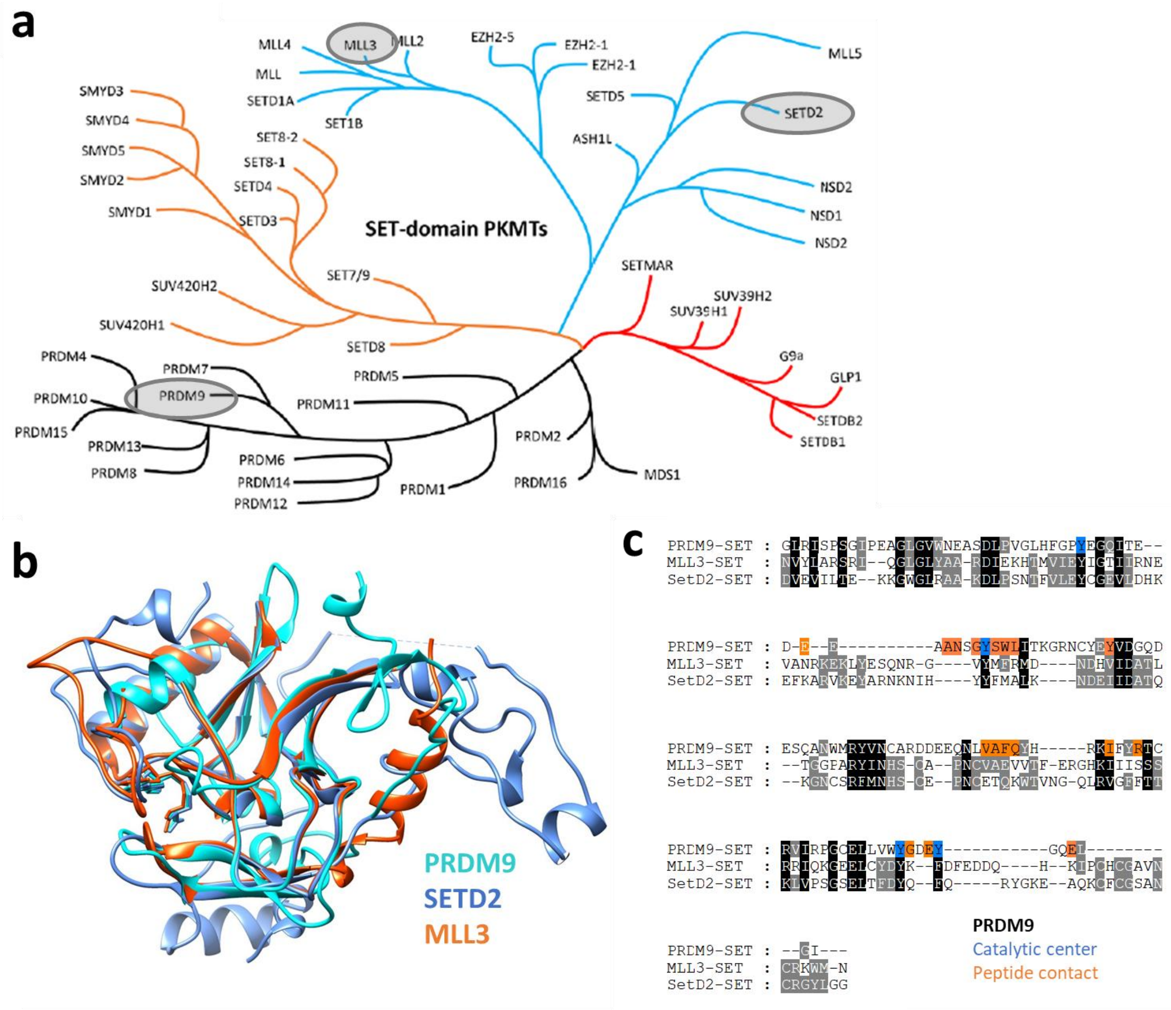

Supplementary Figure 1: Comparison of PRDM9 with H3K4 and H3K36 PKMTs. a PKMT phylogenetic tree taken from Schnee et al. (2024) [1]. PRDM9 as well as MLL3 (a H3K4 PKMT) and SETD2 (a H3K36 PKMT) are highlighted. **b** Structural superposition of PRDM9 (PDB: 4C1Q), MLL3 (PDB: 7W6L) and SETD2 (PDB: 6VDB) generated with Chimera 1.18. **c** Structure based amino acid sequence alignment of the conserved parts of the SET domain of these enzymes (PRDM9 G245-I367). Active site residues are colored blue, residues with peptide contacts in PRDM9 are shown in orange. Note the strong conservation of active site residues but very weak similarities of peptide interacting residues.

Supplementary Figure 2: Additional information related to Figure 1d and Figure 2. a Images of the replicates of the autoradiography of H3K4 and H3K36 specificity scan peptide arrays methylated by PRDM9 (195-415). **b** Distribution of the standard errors of the mean of PRDM9 activity for all H3K4 and H3K36 peptides used for the averaged data shown in Figure 2a and b.

a

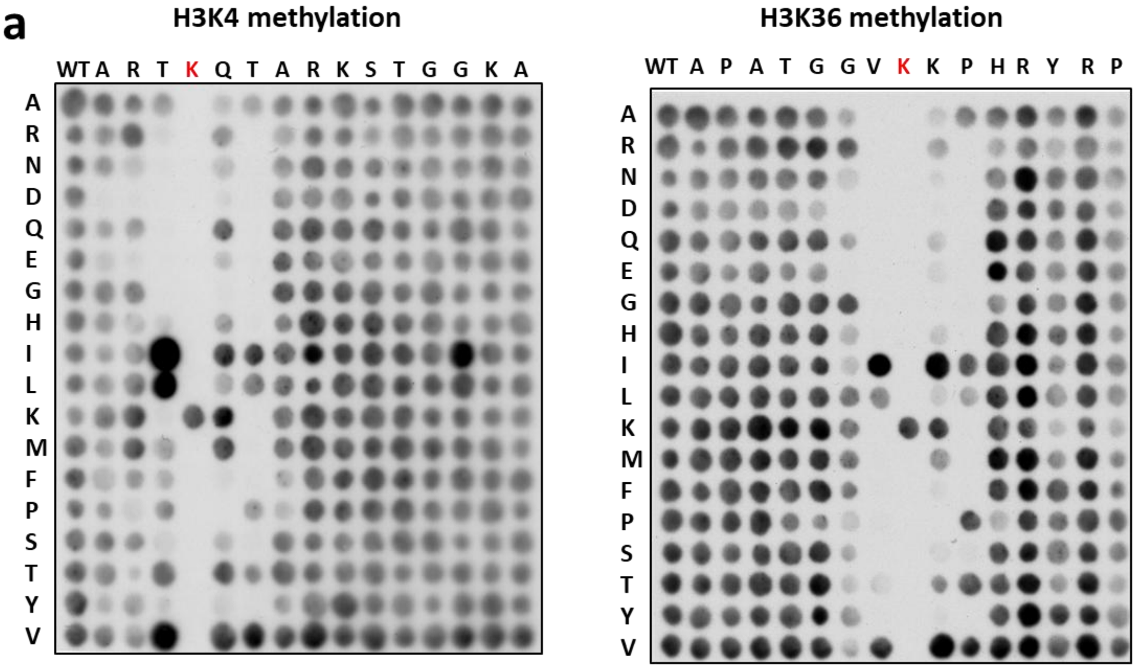

b

H3K4 methylation

Deviation of repeats	Number of Spots
<1%	93
1-2%	31
2-5%	41
5-10%	14
10-20%	1
20-30%	0
30-50%	0
>50%	0

H3K36 methylation

Deviation of repeats	Number of Spots
<1%	77
1-2%	19
2-5%	44
5-10%	24
10-20%	13
20-30%	3
30-50%	0
>50%	0

Supplementary Figure 3: Schematic picture of the setup of the MD simulations.

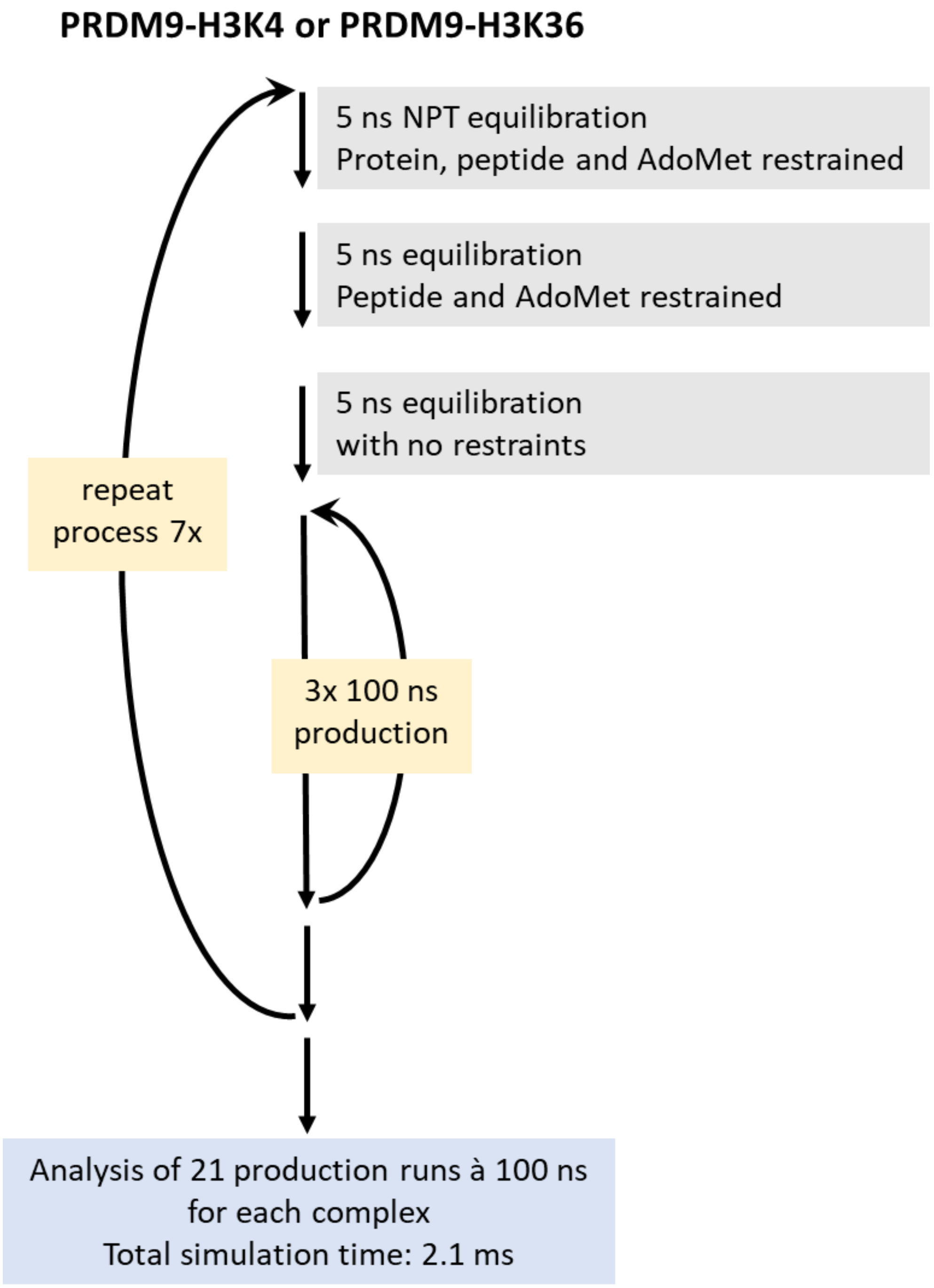

Supplementary Figure 4: Additional information related to Figure 5. a Time course of the methylation of H3K4 (1-19) and H3K36 (26-44) peptides by PRDM9 (195-415) WT using radioactive labelled AdoMet. Exemplary autoradiography pictures showing Tricine gels with the samples from the kinetic experiments performed for H3K4 and H3K36. The quantitative analysis of three experiments is shown in Figure 5B. **b** Peptide methylation experiments conducted in the presence of additional unlabelled AdoMet showing that the H3K4/H3K36 preference is not dependent on the AdoMet concentration.

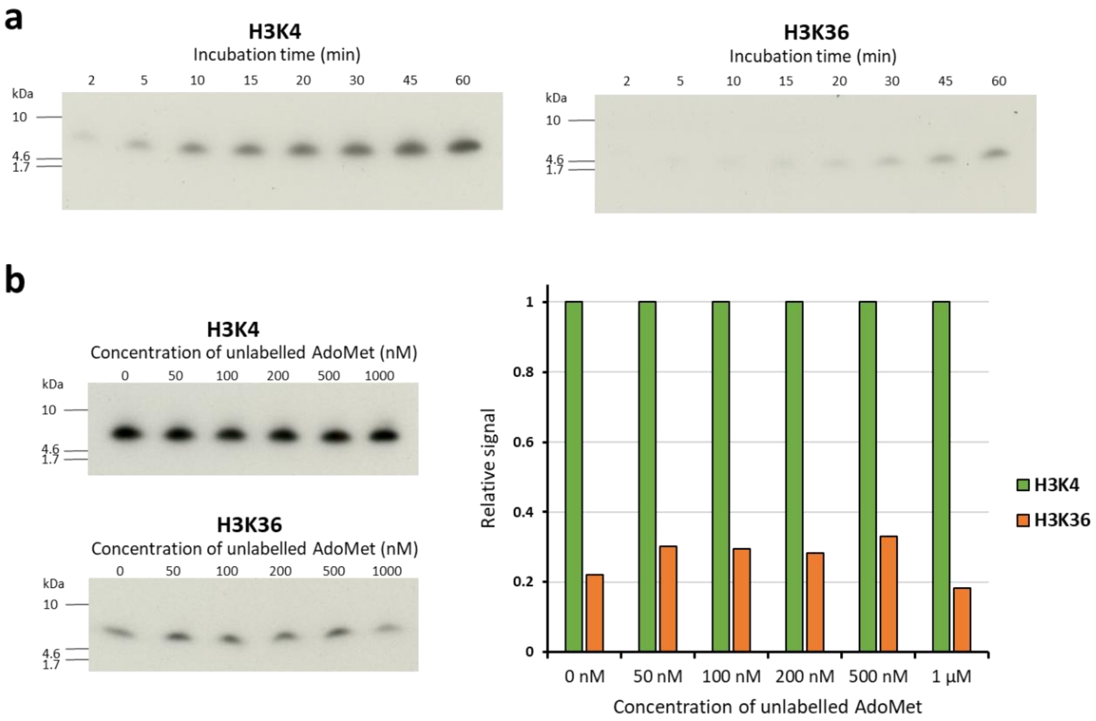

Supplementary Figure 5: Coomassie Brilliant Blue stained SDS gel electrophoresis of purified PRDM9 (195-415) WT and mutants. Corresponding protein bands are marked by asterisks. Note, that loading was different between the different gels, but each gel included a WT protein sample as reference.

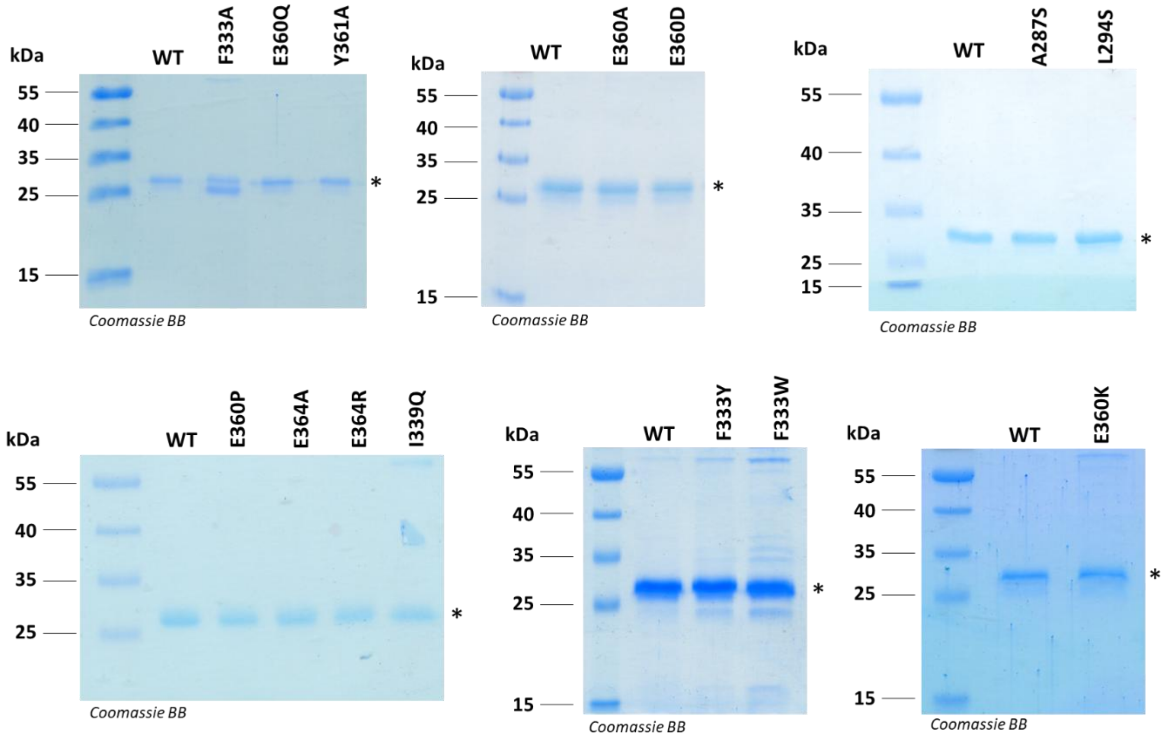

Supplementary Figure 6: H3K4 and H3K36 methylation activity of WT and mutant PRDM9 enzymes. Exemplary autoradiography images of PRDM9 (195-415) wildtype and mutant methylation reactions with soluble H3K4 (1-19) and H3K36 (26-44) peptides separated by Tricine-SDS-PAGE. The methylation reactions were performed with equal amount of PRDM9 (195-415) WT and mutant enzymes and equal concentrations of the H3K4 (1-19) and H3K36 (26-44) peptide substrates.

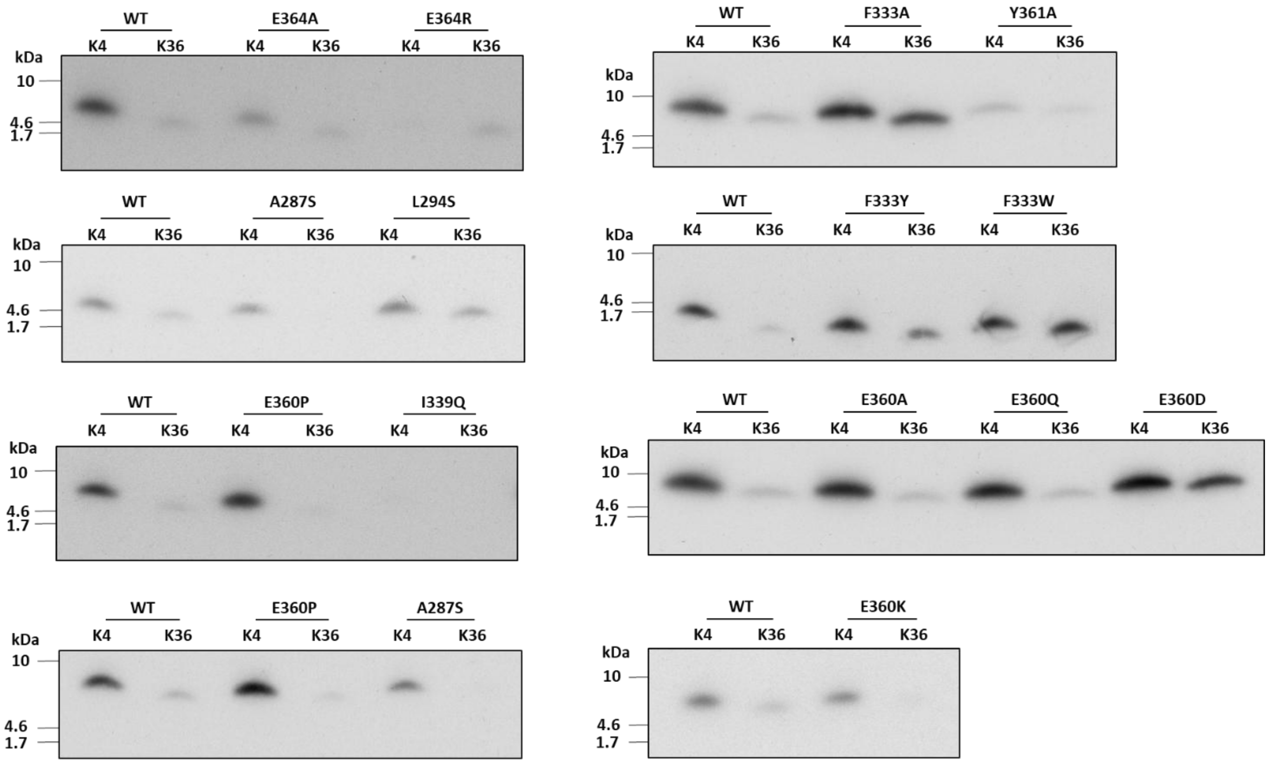

Supplementary Figure 7: Image showing the interaction between the +2 position of the H3K4 peptide and PRDM9 (195-415) derived from the crystal structure (pdb 4C1Q). The residues contacting T6 (L294, F333 and I339) are colored yellow. Residues potentially interacting with the side chain of F333 (F340 and R342) are shown in orange.

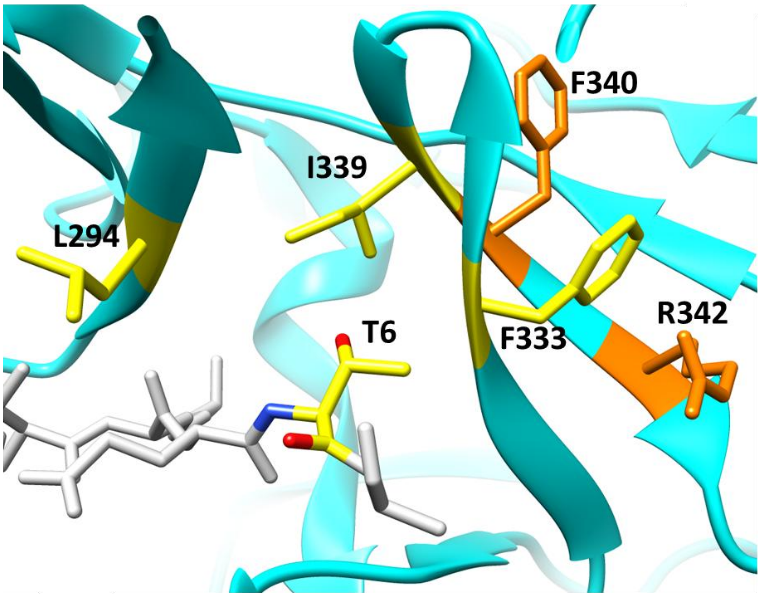

Supplementary Figure 8: Uncropped images of the Figures and Supplementary Figures

Uncropped image of Figure 1b

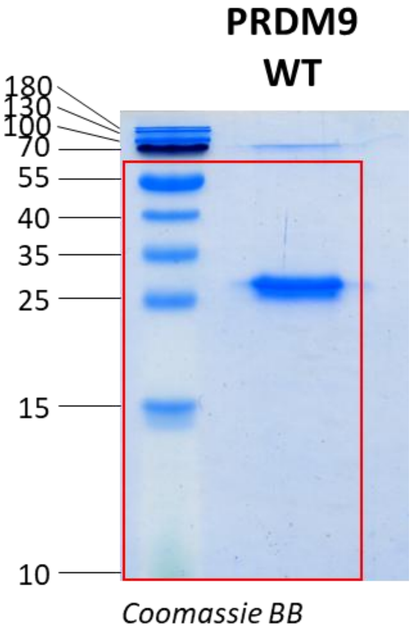

Uncropped image of Figure 5a

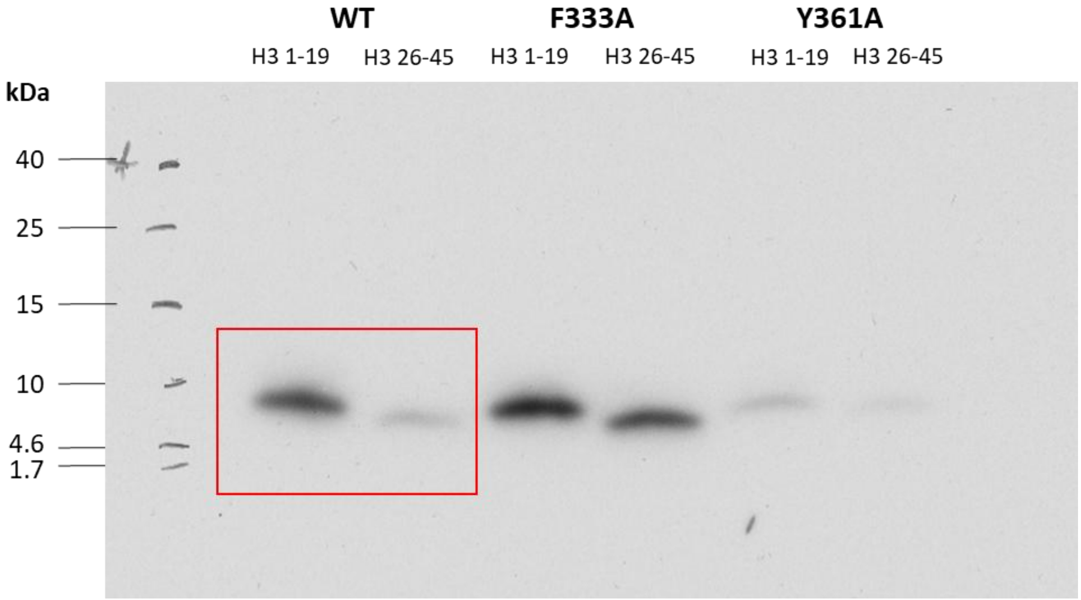

Uncropped image of Supplementary Figure 4a

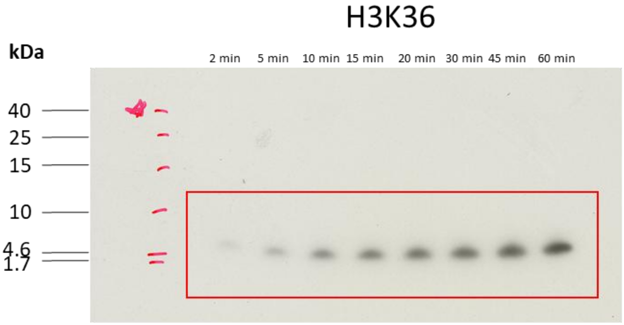

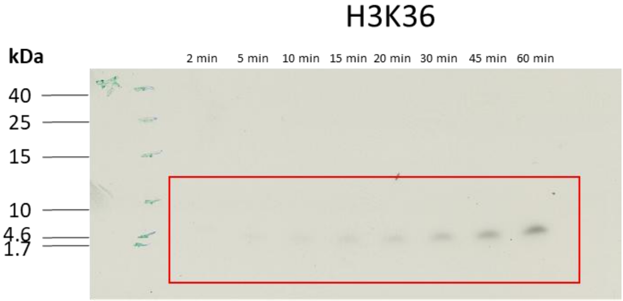

Uncropped image of Supplementary Figure 4b

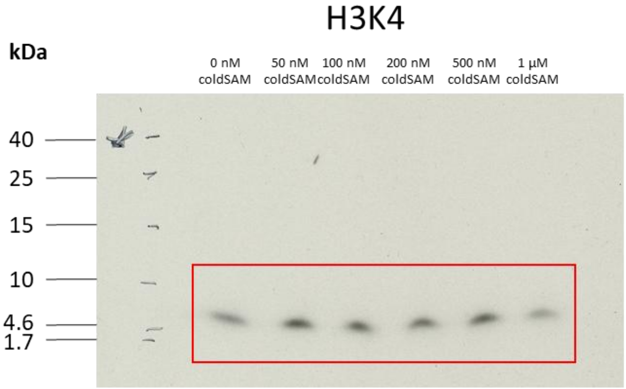

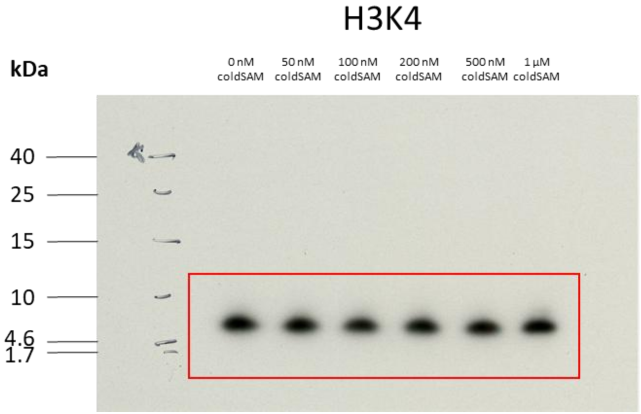

Uncropped image of Supplementary Figure 5

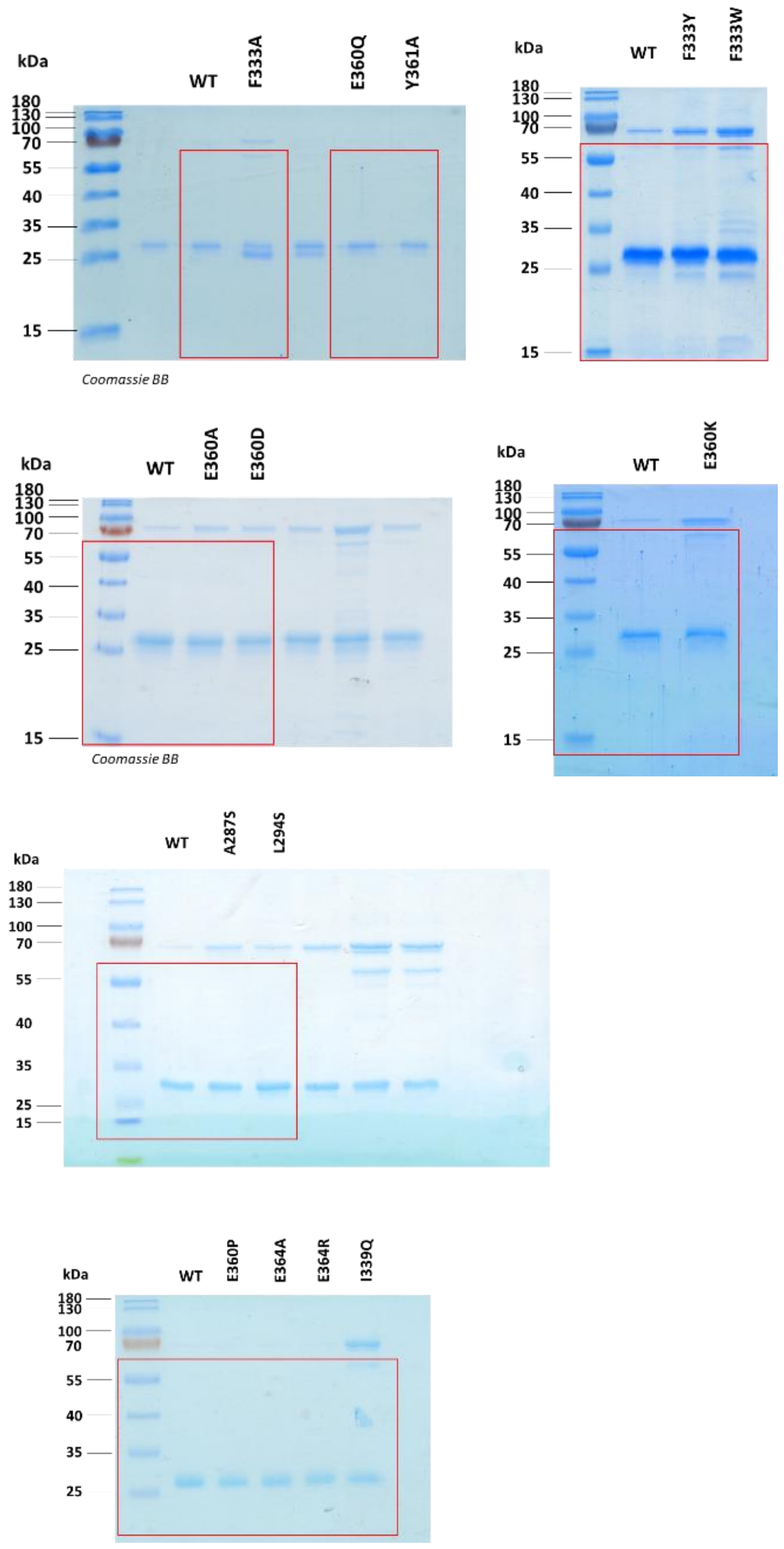

Uncropped image of Supplementary Figure 6

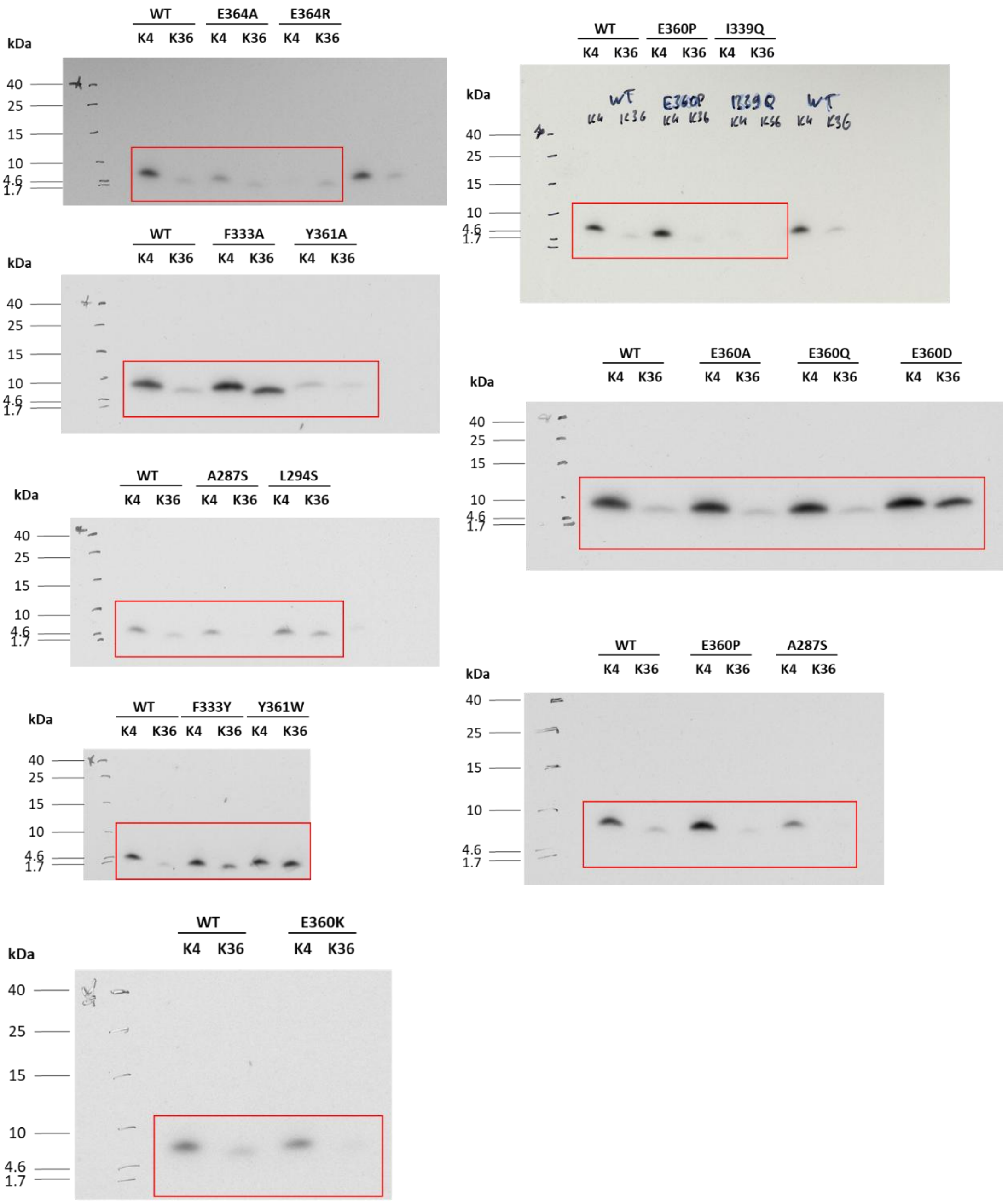

Supplementary references

1 Schnee, P., Pleiss, J. & Jeltsch, A. Approaching the catalytic mechanism of protein lysine methyltransferases by biochemical and simulation techniques. Crit Rev Biochem Mol Biol, 1-49 (2024).
